# New symmetry of intended curved reaches

**DOI:** 10.1186/1744-9081-6-21

**Published:** 2010-04-01

**Authors:** Elizabeth B Torres

**Affiliations:** 1 Psychology Department, Centre for Cognitive Science, Computational Bio-medicine Imaging and Modelling, Rutgers University 152 Frelinghuysen Rd. Piscataway, NJ 08854, USA

## Abstract

**Background:**

Movement regularities are inherently present in automated goal-directed motions of the primate's arm system. They can provide important signatures of intentional behaviours driven by sensory-motor strategies, but it remains unknown if during motor learning new regularities can be uncovered despite high variability in the temporal dynamics of the hand motions.

**Methods:**

We investigated the conservation and violation of new movement regularity obtained from the hand motions traced by two untrained monkeys as they learned to reach outwardly towards spatial targets while avoiding obstacles in the dark. The regularity pertains to the transformation from postural to hand paths that aim at visual goals.

**Results:**

In length-minimizing curves the area enclosed between the Euclidean straight line and the curve up to its point of maximum curvature is 1/2 of the total area. Similar trend is found if one examines the perimeter. This new movement regularity remained robust to striking changes in arm dynamics that gave rise to changes in the speed of the reach, to changes in the hand path curvature, and to changes in the arm's postural paths. The area and perimeter ratios characterizing the regularity co-varied across repeats of randomly presented targets whenever the transformation from posture to hand paths was compliant with the intended goals. To interpret this conservation and the cases in which the regularity was violated and recovered, we provide a geometric model that characterizes arm-to-hand and hand-to-arm motion paths as length minimizing curves (geodesics) in a non-Euclidean space. Whenever the transformation from one space to the other is distance-metric preserving (isometric) the two symmetric ratios co-vary. Otherwise, the symmetric ratios and their co-variation are violated. As predicted by the model we found empirical evidence for the violation of this movement regularity whenever the intended goals mismatched the actions. This was manifested in unintended curved "after-effect" trajectories executed in the absence of obstacles. In this case, the system was "perturbed" away from the symmetry but after several repeats it recovered its default state.

**Conclusions:**

We propose this movement regularity as a sensory-motor transformation invariant of intentional acts.

## Background

The primate arm-hand system has many more degrees of freedom (d.o.f.) than the three-dimensional physical space in which the system operates. Such excess lends primates great flexibility to interact with the external environment and gives rise to highly versatile behaviours. Any goal-oriented task can be performed in a variety of ways. Even under highly constrained laboratory conditions several repeats of the same motion can be highly variable after the motion has been fully automated. To the naked eye every repeat may seem similar to the previous trial but when examined at a millisecond time-scale resolution, different patterns of variability can be revealed. In spite of the many possible ways that exist to perform voluntary reaches, and of the variability of movement parameters manifested in any family of reaching actions, research in the field of motor control has unveiled a number of regular movement patterns that suggest conservation of some underlying quantities possibly driven by sensory-motor integration strategies to achieve some higher-level goal.

Examples abound in motions that are performed under temporal constraints aiming for a particular maximum speed or particular movement duration. Among them the first to be noted was the speed-accuracy trade off known as Fitts' law, discovered by Paul Fitts in 1954 [1]. The law predicts that the time required to rapidly move to a target region depends on the distance D to the target and the width W of the target region, . This law has been reproduced in a variety of situations and more recently extended to the human-computer interaction domain as the Accot-Zhai steering law [2].

Other known regularity of human movements is the symmetric ("bell-shaped") nature of the speed profiles of point-to-point straight motions noted earlier [3] and modelled in two dimensions according to various propositions of what the brain may be optimizing when planning accurate reaches [4-6]. These have included quantities driven by kinematics, quantities driven by force-related parameters as well as by motor noise.

Invariably these studies however have treated the movement time as a free variable and pre-set the duration of the reach a priori. This has also been the case in animal movement research, which has reproduced the speed profile regularity [7,8] in highly over-trained subjects that in some cases have been trained to attain a particular peak velocity within a given time window. This treatment of the motor control problem has left unexplored the question of whether movement regularities could also emerge when the temporal dynamics of intended actions are highly variable. For example we have recently found that during motor learning curved hand trajectories did not conserve the smooth symmetric profiles characteristic of automated straight reaches [9], yet other more subtle patterns may still be conserved under such conditions.

In reach to grasp motions that required the bending and twisting of the hand along the motion paths to match some location and orientation in space, the arm system defaulted to the co-articulation of 4 rotational joints roughly projecting to three physical dimensions for transport of the hand and 1 for rotations of the hand. The subjects performing such motions invariably conserved the relationship between the transport and the rotational errors [10]. This conservation was manifested for different dynamics manipulations, including systematic variations in speed and initial arm posture to induce different muscle patterns and produce different forces. The curves describing such motions have been well characterized as the length minimizing curves (the geodesics) along two related Riemannian manifolds used to align proprioceptive (internally sensed postures) and visual (externally sensed goals) spaces of disparate dimensions and different sensory-processing temporal lags [11].

As one assesses more natural behaviours, movement regularities unveiled in one context do not necessarily transfer to another context. Another known example has been the somewhat controversial human movement pattern known as the 2/3 Power Law. This movement regularity reveals a non-linear relationship between the tangential hand speed and the curvature of its trajectory during curved motions [12-14]. Such relations though appear to be violated in rhythmic three-dimensional arm movements [15], raising the possibility of two separate systems for the control of fast automatic and slow motions. This idea has been supported by clinical research [16,17] and suggested also in the context of motor learning/adaptation studies [18] of planar motions.

Studies involving motor learning may provide a more natural scenario to test the movement regularities described thus far in fully automated reaches, or in motions with specific spatio-temporal requirements. In most experiments describing such phenomena, the arm has been constrained to move on a plane and/or to move under a pre-defined temporal structure that a priori sets bounds on the total duration of the motion and/or on the motion's maximum speed. It is unknown whether the untrained system, moving naturally at its own pace while learning new dynamics would also manifest movement regularities. In natural settings unconstrained motions of the arm would recruit many of the rotational d.o.f. in the proprioceptive space of sensed postural configurations and the motions would generally be more variable. Would other subtle invariants emerge under such conditions? More importantly, are there motion invariants that remain conserved across different families of reaching actions? Under what conditions such invariants would be violated and repaired? We address these questions here in two untrained monkeys as they learned to avoid obstacles when their hands aimed at visual targets in the dark.

## Materials and methods

### Experimental methods

**Figure 1:**
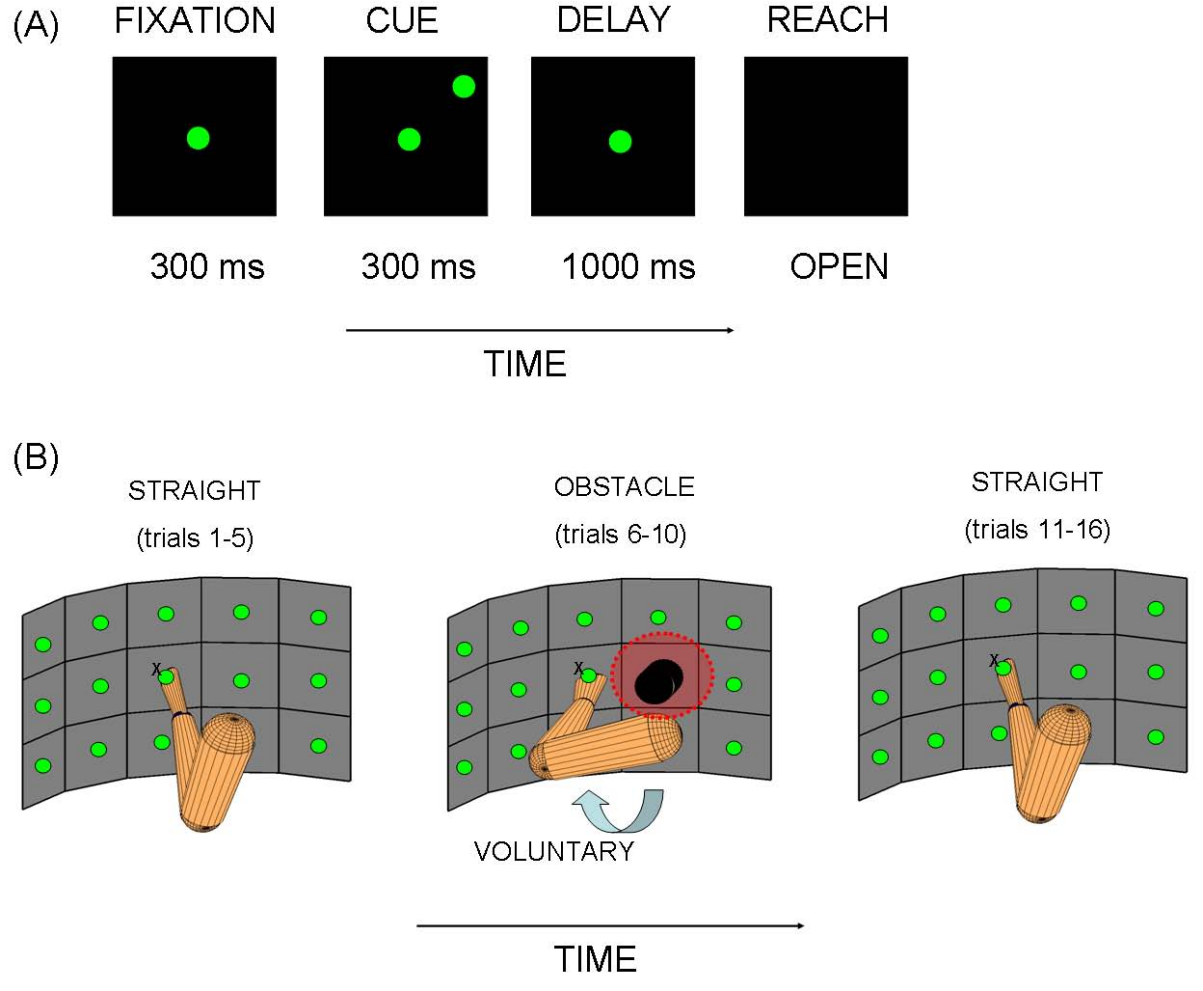
**Experimental paradigm designed to naturally evoke the learning of obstacle-avoidance**. (A) Temporal epochs and visual stimuli in each single trial of the experiment. The monkeys were neither conditioned to end the reach at a particular time nor to reach a particular velocity peak. They moved at their own natural pace. (B) Block-design experiment to naturally evoke learning consisted of straight reaches, followed by new reaches around physical obstacles for which the monkeys had not been trained, and then straight reaches again. Red circle marks the site of maximal memory response in the visual field from a posterior parietal neuron used to guide the initial placement of the obstacle. The second placement was on the opposite side of the board.

Two rhesus macaque monkeys were first trained to perform memory-guided straight reaches directed to visual targets in the dark. Targets were located on a vertically-oriented board (figure 1B). A block-design paradigm was used to interleave such automated three-dimensional reaches with reaches for which the animals had not been trained. These consisted of reaches to the same targets on the board but performed to avoid physical obstacles (OB) in various configurations. The OB(s) were placed on the board blocking the straight path to some of the targets and evoking variable degrees of hand path curvature. The monkeys performed the task with their right hand.

The structure of the paradigm was A-B-A', where A represented the automated straight reaching block, B was the block of curved reaches around obstacles and A' was the last block of straight reaches after exposure to many highly fast and curved OB-avoidance-motion repeats. The experimental paradigm and other aspects of the learning process have been described elsewhere [9]. Here we focus on geometric measures of the hand trajectories.

Since these experiments monitored the two animals over several months until their new reaches turned automated, here we focus on the time period when the animals were still learning. The criteria for learning vs. automatic came from (1) the jerkiness vs. smoothness of the new speed profiles along the curved hand paths, and (2) the ease with which the animals switched from one experimental block to the next. The presence of jerky hand speed profiles when switching from A to B, or from B to A' indicated that the OB-avoidance motor program was still fragile, i.e. undergoing learning [9].

Since our main interest rests on the effects that the high variability inherent in the learning process may have on movement regularities, we will focus on the behavioural data from the earlier days of training. The data was gathered from experimental sessions where the transition from A to B, or from B to A' was not direct. In those days it took many trials to transition from straight paths with smooth speed profiles to curved paths which would also develop smooth speed profiles (going from A to B); or it would take many trials to transition from curved paths with smooth speed profiles to straight paths with smooth speed profiles (going from B to A'). In particular, in the transition from B to A' unintended curved hand trajectories in the absence of OB were coined "after-effect" trajectories from OB-avoidance and we studied the evolution of those trajectories in depth here.

The motions of the performing arm were recorded using electro-magnetic sensors (Polhemus Fastrak 120 Hz resolution). The hand sensor was mounted on a piece of Plexiglas, affixed to a custom-made glove and placed on the surface of the moving hand, one cm from the wrist line. The rest of the sensors were mounted on a piece of Plexiglas and affixed to a primate jacket at the shoulder, upperarm and forearm.

The monkeys were seated in a primate chair. Since concurrent neural recordings were being obtained, their head was not moving and their eyes were fixating straight ahead. They moved their arm freely in three dimensions and received a juice reward after each successful trial. Successful trials had the following structure (depicted in figure 1A): in complete darkness, after the animal fixated to a light straight ahead for 300 ms, a target light was flashed for 300 ms. A memory period variable between 800-1000 ms ensued when the animal had to withhold the reach and maintain fixation until the GO signal. The GO signal was the offset of the fixation light. A reach period followed when the animal had to achieve the memorized target location in complete darkness. In the OB-avoidance block the same structure was used but the animals viewed the OB(s) prior to the initiation of the block, when the lights in the room went off, and they had to memorize the OB(s)' location and configuration in order to avoid them.

The data discussed here is from avoiding obstacles that were cylindrically shaped black blocks protruding 5 inches out of the board and measuring 3 inches in diameter. The locations of the obstacles were primarily on the right or on the left sides of the central fixation point on the board. Since the main goal of this study was to investigate the role of the Parietal Reach Region cells on changes to postural trajectories from curved motions, these locations were chosen according to the cell's visual receptive field (the OB were placed in and out of it). The present study focuses on locations to the right and to the left of the fixation point as these evoked the highest changes in curvature and timing.

The subjects were not penalized if the hand collided with the OB(s), as we were interested in the evolution of such trials. However, not enough trials where they hit the OB(s) could be gathered for analyses as both animals were naturally very proficient at successfully avoiding the OB(s) while maintaining eyes fixated in the dark. The data reported here comes from trials where no collision with the OB(s) occurred.

All experimental procedures were conducted according to the "Principles of laboratory animal care" (NIH publication no. 86-23, revised 1985) and were approved by the California Institute of Technology Institutional Animal Care and Use Committee.

### Behavioural and statistical measures

#### Bending and twisting of the hand paths

We assessed the effects of task condition and target location on the curvature of the hand trajectories. To this end we measured the deviation of the hand trajectory from the Euclidean straight line and used this as a bending index (denoted κ).

The starting and final location of each hand trajectory was joined by the corresponding straight line. The hand trajectories were re-sampled (100 points) to obtain a fine temporal partition at equally spaced points without distorting the spatial path. This was necessary for numerical integration to compute the area and the perimeter ratios explained below and to treat the curve as a geometric object independent of its temporal profile. The latter is justified by empirical evidence indicating that during voluntary motions, primates conserve the hand paths regardless of the motion's speed [9,11,19-21]. Points in the re-sampled hand path were projected onto the straight line and the normal distance from each point along the curve to the corresponding point on the Euclidean straight line was measured to determine the point of maximum normal distance (maximum bending κ) in each path.

**Figure 2:**
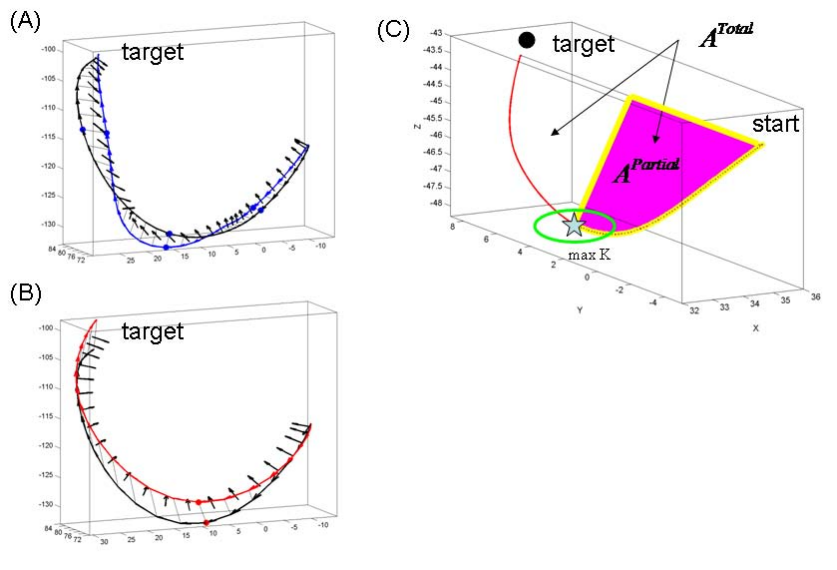
**Trajectory geometric measures of interest**. (A) Twisting the line is defined by the angle between two consecutive vectors normal to the plane spanned by projecting the acceleration vector onto an ideal geodesic curve. Blue trajectory is jerky (segmented) from the learning data, black trajectory is modelled (see Additional file 1). Blue dots mark the critical points (speed maxima) of the segments. (B) Same twisting measure in the smooth automated case. Red dot is the single velocity peak. Black is the modelled path. (C) Ratios of interest: For each hand trajectory the curve was re-sampled at equally spaced intervals. The points along the curve were projected on the Euclidean straight line joining the start and target locations. The point of maximum bending was obtained and the area enclosed between the lined and the curve computed and denoted partial area *A*^Partial. ^To obtain the area ratio this quantity was divided by the total area enclosed between the curve and the line, denoted *A*^Total^. The perimeters were similarly obtained using the length of the curves instead.

We also measured the degree of twisting along the hand paths. At each point along the trajectory the tangent vector to the curve (the velocity vector) and the vector perpendicular to it (the acceleration vector) span a plane (known as the osculating plane [22]). This plane changes as the hand changes position in time. The tilt of the vector **normal **to this plane at each position was compared to that corresponding to the plane from the previous position in order to measure the twisting of the curve along the hand motion's trajectory (i.e. given two consecutive tangent vectors and corresponding orthogonal acceleration vectors, we obtained the angle between the two consecutive vectors normal to the two osculating planes). To better visualize this relative to an ideal OB-avoidance modelled geodesic we show in figure 2A, B the projection of the data trajectory on the geodesic and mark the normal to the plane spanned by the projection. As in the bending case, this progression gave the twisting profile of the motion as a function of time. Then we obtained the point in time where the torsion of the hand path was maximal (measured in degrees). (Notice that we chose not to use the Frenet-Serret formulas to formally compute curvature and torsion of the curves to avoid numerical errors in the computation of higher derivatives from the sampled data).

Two-way ANOVA (target location and experimental condition as the two factors and κ (or T) as the dependent variable) was used to assess significance in the differences across experimental blocks. Within each block we also performed an ANOVA to ask if the target location had a systematic and significant effect on the bending (or on the twisting) parameter.

#### The relative timing of critical kinematics temporal landmarks

We measured in each trajectory the timing of several kinematics parameters occurring at different points of the motion. These critical temporal markers were: the *time length to reach the maximum speed *from the start of the reach (denoted tau); the *time length to reach the maximum acceleration *(denoted α) and the total duration (denoted *t*). In particular we were interested in the evolution of the magnitude of the acceleration relative to the magnitude of the velocity. *Where *and *when *along the curve traced by the hand were these quantities maximal? Was there any evidence that the system was using a particular critical temporal point as a reference while timing the reaches?

We had previously reported that the speed profiles during the learning phase of the new OB-avoidance motion were jerky, with multiple accelerations and decelerations phases and highly variable in duration ranging from 1,500 ms to 700 ms for the same target [9]. Motivated by the different force patterns required to complete these reaches with strikingly different tempo, and by the conservation of the postural and of the hand paths, here we asked if there were fundamental differences between the temporal acceleration profiles of these two different families of reaching trajectories -straight and curved-as the subjects built a new procedural memory. Notice that we are using the magnitudes of the velocity and of the acceleration (a positive scalar).

To assess the effects of target-dependent curvature/torsion and learning on tau, α and *t *we used two-way ANOVA with target location and experimental condition as the two factors, and the temporal-dynamics parameters as the dependent variable in each case.

#### Trajectory ratios, symmetry and similarity

We defined two trajectory ratios:

(1) The **area ratio **was defined as the quotient between the partial area under the curve in the first portion of the movement, up to the point of maximum bending κ, and the total area enclosed between the curve and the line. Figure 2c illustrates the definition.

(2) The **perimeter ratio **was defined as the quotient between the partial perimeter -the sum of the path length and the length of the line connecting the initial hand position and the target up to the point of maximum bending- and the total perimeter given by the total sum of the lengths of the hand path and the initial hand position-to-target line.

It is important to note that the assessment of these quantities in the context of motor learning was motivated by a previous finding involving human subjects. In that study humans performed similar three-dimensional point-to-point straight reaches under visual memory guidance in the dark. Both aforementioned ratios were time-invariant, i.e. remained conserved for multiple speeds along paths of similar curvature. The conservation was in that case also independent of the frame of reference used to cue visual guidance [23]. This was also a theoretical result [11] explained through a simple case in **the Appendix **and qualitatively congruent with previous data from various laboratories showing the conservation of voluntary arm postural and hand paths [9,10,19-21] in the face of highly variable timing and different loads applied to the arm.

The novelty of the questions addressed in the present experiments rests in two facts: (1) the animals were untrained to perform the task of interest; (2) there were dramatically different arm force patterns to achieve multiple families of curved hand trajectories.

#### Similarity of the area and perimeter ratios

We obtained from each hand trajectory the area and perimeter ratio and used the Friedman test [24] to assess their similarity in each experimental condition. We addressed the effects of hand path curvature and temporal dynamics on these quantities for all experimental conditions: A (automatic), B (intentional learning of new speed profiles) and A' (de-adaptation as the special case where curved hand trajectories emerged unintentionally when obstacles were no longer present and the intended goal was as in A, to move straight to the target).

## Results

### Bending and twisting of the hand paths

**Figure 3:**
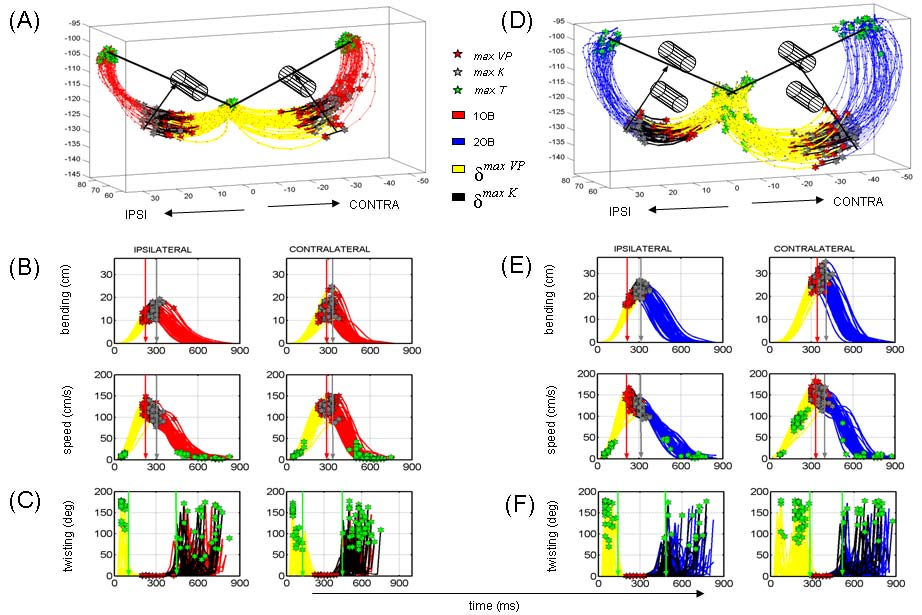
**Spatio-temporal parameters in different families of curved motions**. (A) Hand trajectories Ipsi- and contra-lateral to the performing arm from motions avoiding 1OB. Yellow denotes the distance travelled up to the first velocity peak (red star), black is the distance travelled up to the point of maximum bending (grey star) and the green star denotes the point of maximum twisting. Notice that these points of high torsion are detected rather at the earliest or at the latest in the path during avoidance of 1 OB. (B) Bending and speed profiles as a function of time with arrows marking when along the hand path the maximum bending, speed and twist are reached. (C) Hand twisting profiles as a function of time. (D-F) Similar plots for significantly more curved and significantly faster hand trajectories avoiding 2 OB(s). Notice that unlike in the less curved trajectories, some of the points of maximal torsion are reached midway in the acceleration phase of the path.

We found significant effects of target location and experimental condition in both animals (alpha 0.01) on the bending and twisting of the hand trajectories. In the straight-reaching block the target location affected both trajectory parameters significantly. These results extended from the straight-reaching to the OB-avoidance block. There was a significant effect of the block-condition. Figure 3 shows typical ipsi- and contra-lateral trajectories to the performing arm from one of the animals, along with the bending, twisting and speed profiles for avoiding 1 or 2 OB(s). We found in both animals that the curvature and torsion of the hand paths significantly depended on the target location, on the number of OB(s) and on the learning stage (block). For less curved motions twisting was confined to the initiation and ending of the reach, and the main movement segment remained fairly planar. However, as the curvature increased, the hand twisted more along the path (figure 3D, E, F). These interaction effects were significant at 0.01-level according to the 2-way-ANOVA in both animals. Table 1 lists the average values for various spatio-temporal parameters from the two animals and the results of the ANOVA test.

**Table 1:** Parameters of interest measured in the 2 monkeys

Targets	AVRG	1	2	3	4	5	6
***Max VP *(cm/s)**	F = 19.2, p < 10^-5	116 ± 10.5	84.8 ± 5.2	87.1 ± 7.9	111 ± 12.9	85.4 ± 10.7	83.4 ± 8.2
		136 ± 53.3	130 ± 7.9	125.4 ± 4.5	141 ± 18.6	137.3 ± 17.2	130.9 ± 6.8
		151 ± 19.8	148 ± 23.3	143.8 ± 3.4	159 ± 40.5	150.8 ± 17.8	145.6 ± 27.6

***Max K *(cm)**	F = 22.2, p < 10^-7	6 ± 1.1	2.8 ± 1.0	1.2 ± 0.6	5.9 ± 1.18	3.05 ± 0.9	2.21 ± 0.7
		14 ± 2.3	6.83 ± 2.5	2.9 ± 2.3	15.2 ± 3.0	8.9 ± 2.2	5.6 ± 1.8
		23 ± 2.1	11.7 ± 5.13	7.4 ± 3.1	22.8 ± 6.4	19.9 ± 2.9	9.7 ± 4.7

***Max T *(deg)**	F = 22.1, p < 10^-7	52 ± 17.2	47.8 ± 17.8	43 ± 16.2	46.6 ± 12.8	46.4 ± 18.8	46.1 ± 20.3
		116 ± 53.4	119 ± 47	105 ± 38	118 ± 32.5	118 ± 48.1	115.5 ± 53.4
		139 ± 35.1	133 ± 40	108 ± 44.	142 ± 27.9	128.3 ± 30.9	140.0 ± 32.4

Δδ **(cm)**	F = 9.2, p < 10^-4	4 ± 1.8	1.8 ± 2.4	1.03 ± 1.4	1.82 ± 0.6	-0.5 ± 3.1	-2.3 ± 3.5
		7 ± 5.18	3.1 ± 3.4	2.06 ± 1.9	1.15 ± 1.4	-2.2 ± 1.6	-4.2 ± 0.5
		12 ± 2.3	8.6 ± 3.4	4.87 ± 2.9	3.8 ± 0.3	-9.7 ± 2.3	-12.2 ± 2.2

**tau (ms)**	F = 17.2, p < 10^-6	235 ± 41.8	228 ± 39.2	216 ± 18	251 ± 41.3	231.9 ± 24.1	211.3 ± 39.8
		237 ± 39.3	237 ± 37.8	223 ± 17	292 ± 37.5	241.4 ± 23.9	245.9 ± 34.9
		246 ± 34.9	243 ± 29.0	239 ± 3	344 ± 33.2	314.4 ± 31.1	303.3 ± 27.5

	F = 7.7 p < 0.001	2 ± 0.3	2 ± 0.1	2 ± 0.2	2 ± 0.3	1.9 ± 0.2	2.0 ± 0.4
		2 ± 0.2	2 ± 0.2	2 ± 0.2	1.9 ± 0.2	1.8 ± 0.2	1.7 ± 0.1
		3 ± 0.4	3 ± 0.3	3 ± 0.3	2 ± 0.2	1.7 ± 0.2	1.7 ± 0.3

Parameters were averaged across data from 2 subjects for automated straight reaches, reaches avoiding one obstacle and reaches avoiding 2 obstacles. Parameters included the maximum velocity peak (out of multiple peaks) in cm/s, the maximum bending (κ in cm), the maximum twisting (T in degrees), the difference in distance travelled delta between the point of maximum bending and the point of maximum speed along the hand path (in cm)-negative values indicate that the peak bending occurred prior to the peak velocity; the time tau to reach the first velocity peak (in ms), and the unit less ratio of total hand path length (cm) to distance travelled to the first velocity peak (cm) -a value of 2 means that the speed profile was symmetric. The 2way-ANOVA results (averaged F-value and Prob>F across the 6 most affected targets and 2 monkeys) are displayed in the first column.

### The relative timing of critical kinematics temporal landmarks

**Figure 4:**
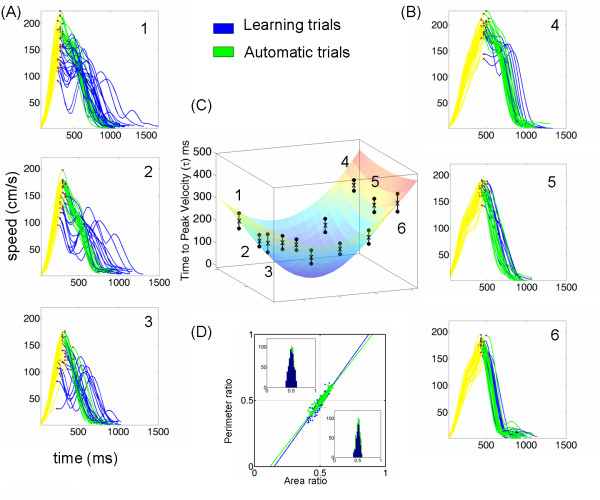
**The dynamics-invariant nature of tau in the first pulse of the reach**. (A) Monkey speed profiles from hand motions to targets 1-3 contra-lateral to the moving arm and 4-6 ipsi-lateral to the moving arm. For each target, trials are colored as learning (blue) and automatic (green) in the same order in which they were recorded. Criteria for automaticity were consistency in the distance delta travelled up to the first velocity peak, a single velocity peak and consistency in the total duration t. Yellow marks the acceleration phase (first pulse of the reach) with a highly variable distance delta travelled at a consistent time tau. Dots mark the first velocity peak. Notice that unlike point-to-point straight reaches these curved reaches have skewed speed profiles. Their acceleration phase is shorter than their deceleration phase in the contra-lateral targets. (B) Ipsi-lateral targets 4-6 have a longer acceleration phase. (C) Mean tau values at each measured location +/- two standard deviations from the mean. Twelve out of 15 locations were used to fit a surface through the points using Matlab interpolation scheme (central location was the fixation point and the other 2 missing points were blocked by obstacles). (D) Trajectory time-invariant area and perimeter symmetries were not affected by the learning of the new dynamics or by the skewed shape of the speed profiles.

*The time to reach the first velocity peak*: Each target location in space had a characteristic value of tau specific to that location in each task. This parameter was highly consistent at each location despite the fact that in each experimental block, the repetitions of the reach were made to randomly presented targets. Figure 4 shows the distribution of tau across space for the case of two OB(s). We fitted a surface through the mean values of tau and plotted the two standard deviations from the mean value at each location where the bending of the hand trajectory was most significant. Notice for each target location of figure 4 the invariance of tau to the dramatic changes in tempo. During the learning trials, notice in figure 4 the contrast between the consistency of tau and the high variability of the total movement duration *t*. This result extended to other families of reaches in the present study.

**Figure 5:**
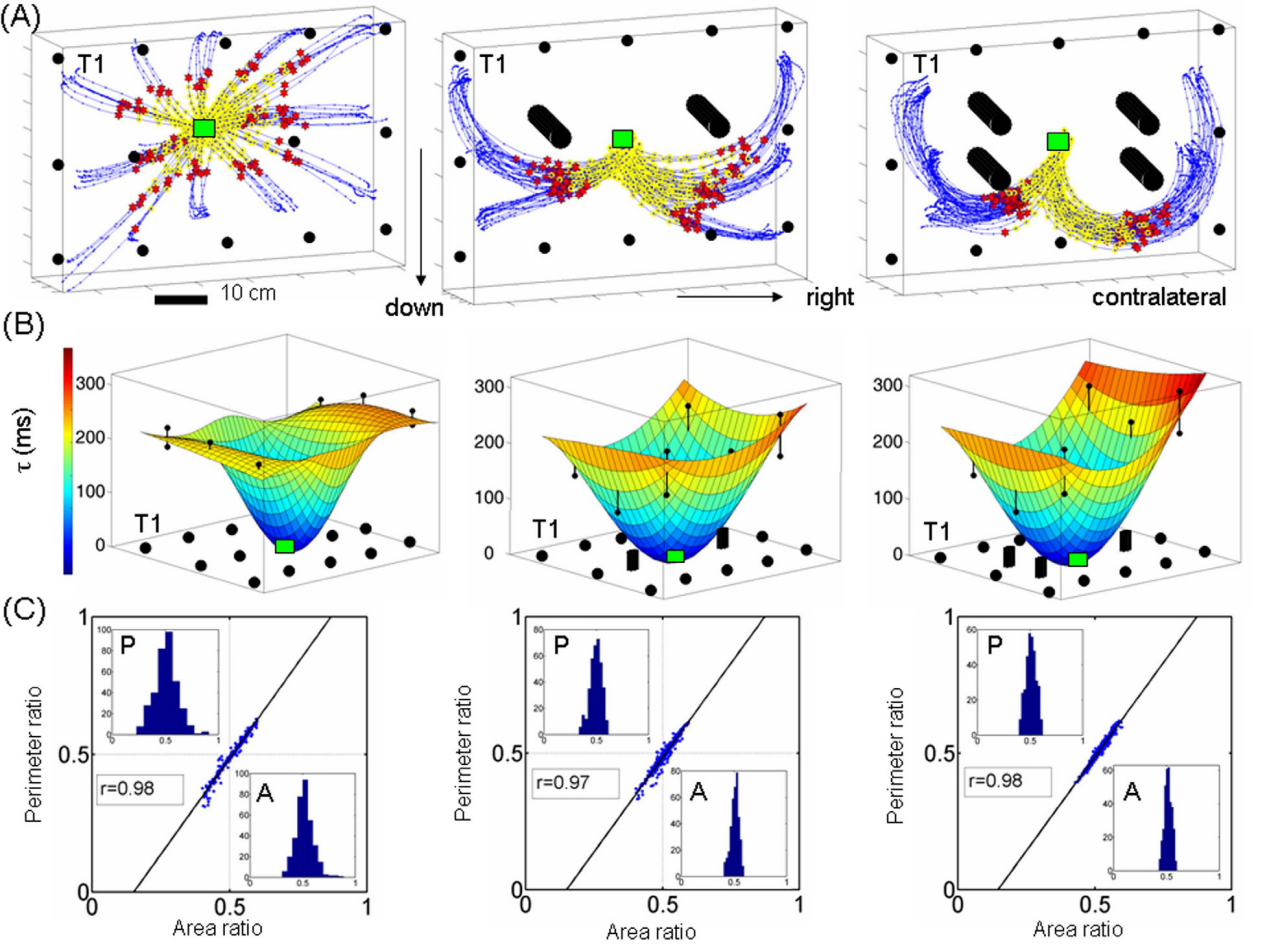
**Geometric invariant at the crossroad of time and distance**. Typical hand trajectories to targets in space with and without obstacles pooled from experiments in one day of early training (from one subject and similar to the other). Initial acceleration phase highlighted in yellow. (B) Fitted surfaces to obtain the mean temporal maps (tau ms) across space to reach the first peak velocity (red dots) on the way to targets in (A). On the xy-plane we represent by dots the locations of the targets and by cylinders the location of the obstacle(s). The distribution of tau reveals the underlying distribution of path length scaled by path curvature. (C) Time invariant geometric ratios (each point is from a trajectory) significantly co-varied. The similarity of the area and the perimeter ratios was confirmed by the Friedman's test [27] with the slopes and the intercepts of the regression lines as ([*1,39*, -*0.21*, [*1,38*, -*0.20*], [*1,36*. -*0.20*]) for straight, 1 obstacle and 2 obstacles respectively.

**Figure 6:**
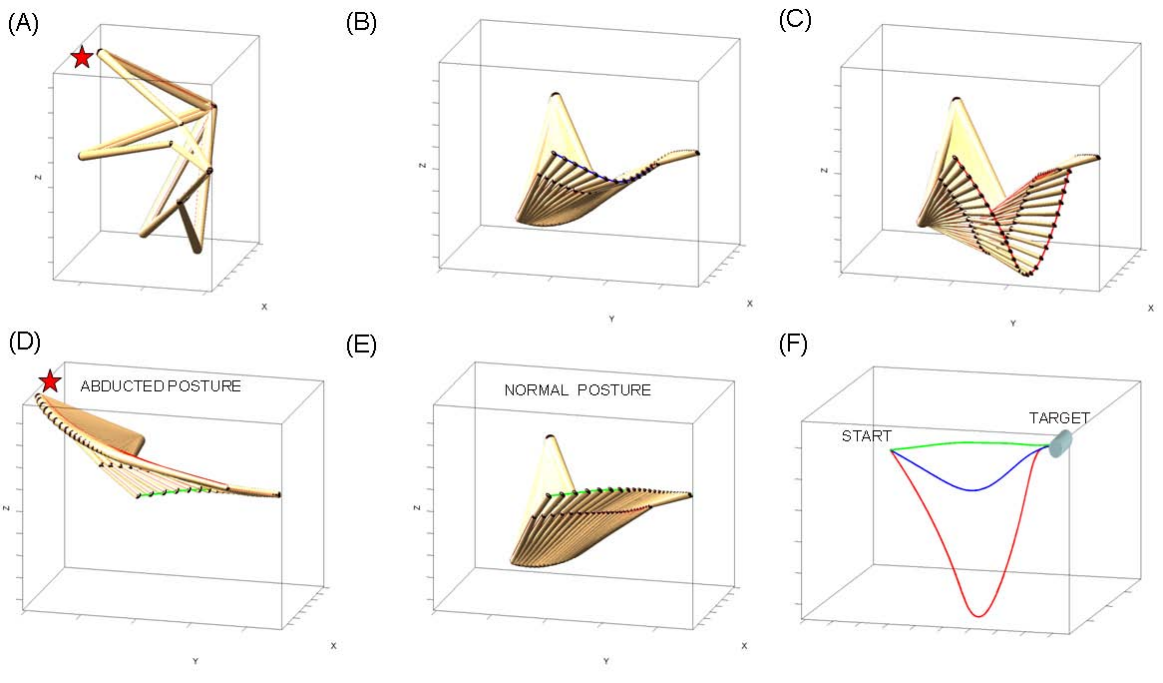
**Gradient-flow-driven distance-metric preserving transformation from intended goals to postural configurations in a more realistic scenario (see appendix 1)**.

Figure 5 depicts the performance of the same animal for the cases of straight reaches, reaches around 1 OB and reaches around 2 OB. In each case the distribution of this temporal parameter tau was predictive of the underlying distribution of path lengths scaled by hand path curvature. As the length and curvature of the path increased the tau-distribution across space orderly changed with a monotonically increasing trend for targets located across the body (contra-lateral to the performing arm) and a monotonically decreasing trend for ipsi-lateral targets. We had previously used different distance metrics, each specific to each tau-family to characterize this phenomenon [9] (see also the figure 6and appendix 1) illustrating length minimizing curves with respect to these different distance-metrics for 3 different space curvatures). The performance of the other subject was also consistent with this one's.

*The time to reach the maximum acceleration*: The temporal profiles of the *magnitude *of the acceleration revealed that the hand trajectories in general had several acceleration peaks. We obtained the percentage of the total movement duration time that it took to reach the absolute maximum acceleration for each condition and plotted the distribution of this parameter across all target locations and reach repetitions. Since the total length of the path relates to the overall duration of the reach, we also examined the distance delta travelled to the first velocity peak, as the hand completes the first pulse of the reach.

In straight reaches the distribution of the percent of time that it took to reach the absolute maximum acceleration along the curve turned out to be strongly bimodal according to the Hartigan's dip test [25,26] for bimodality (*p < 10^-6, dip = 0.09*). A mixture of Gaussians fit yielded 55% of trials with the maximum acceleration reached earlier at 10% of the total time and 45% of the trials with the maximum acceleration reached later at 20% of the total time.

**Figure 7:**
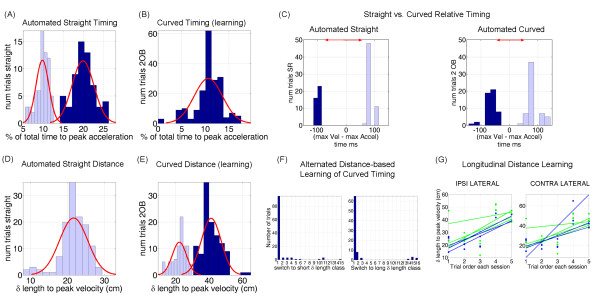
**Hand trajectories: Straight vs. curved timing and distance**. (A) Automated straight timing had two trial-clusters according to the percentage of total movement time to attain the maximum magnitude of the acceleration vector. In one trial-class the hand accelerated maximally much earlier than in the other. Across spatial targets trials lasted 500-700 ms. (B) Learning the curved timing turned this distribution unimodal. (C) Automated straight timing showed two clusters of trials according to the relative time difference between velocity and acceleration maxima. Negative values indicate that the maximum magnitude of the acceleration was reached before the maximum speed. Automated curved timing trials lasted 700-850 ms, spread out the clusters and shifted (contracted) the time-length relative difference between speed and acceleration maxima. (D) Straight path had a unimodal uniform distribution of distance (delta) travelled to the peak velocity. (E) Curved-distance learning turned this distribution non-unimodal. (F) Alternating adjustment strategy of delta in curved-timing learning. If in trial n the hand would travel a short delta, most probably in trial n+1 the hand would travel a long delta. (D) When grouping consecutive trials in one day a monotonic trial-by-trial trend was revealed with an increase of the delta adjusted from one trial to the next. This trend was also observed across consecutive days of training during a week. Blue are from the (multi-peaked speed) learning phase and green are from the (single-peaked speed) automated motions.

Further analyses of all trial-times in each class revealed that each class of trials was composed of reaches to all target locations (uniformly distributed across space) and trials performed uniformly at all times (early and late) in the block. These results ruled out a possible effect of target location preference (e.g. ipsi-vs. contra-lateral or upward vs. downward preferences) or an effect of fatigue (early vs. late trials in the block). This bi-modal distribution defined two trial classes in the straight reaches, one in which the peak acceleration preceded the peak velocity and the other in which the peak acceleration followed the peak velocity. The distribution of time stamps in these trials revealed an alternating strategy whereby it took mostly one or two trials across all repetitions to switch from one trial class to the other. This is depicted in figure 7f. We discuss potential reasons for this performance strategy later.

The OB avoidance block yielded a very different picture with a unimodal uniform distribution of the percent of the total time that it took to reach the maximum acceleration α. This was the case when avoiding 1 (Hartigan's *dip = 0.03, p = 0.6*, failed bimodality test) as well as 2 OB(s) (Hartigan's *dip = 0.01, p = 0.2*, failed bimodality test).

The distribution of delta was also different when comparing the procedural straight reach and the new OB-avoidance reach. During automated straight reaches, the trial distribution of delta was unimodal and uniform across targets (failed the Hartigan's bimodality test, dip = 0.03; p = 0.5). Avoiding 1 OB also failed the bimodality test (dip = 0.02, p = 1). However learning to avoid 2 OB(s) yielded a significantly non-unimodal distribution (Hartigan's bimodality test, dip = 0.09, p < 10^-6). Figure 7a, b shows the distributions of the percentages of time to maximum acceleration and the distributions of the portions of the path length travelled up to the maximum velocity for straight vs. highly curved OB-avoidance around two obstacles. Notice the complementary differences between fully automated straight reaches and highly curved reaches around 2 obstacles when the system was still undergoing learning of the new curved timing. As in the α-bimodal distribution, each of the 2 classes of the delta-bimodal distribution was composed of trials to all targets and time-of-occurrence in the experimental block. Figure 7c shows the distributions of the relative timings of speed and acceleration maxima for fully automated straight and curved timings. Notice the structure similarity to the straight timing inherited by the curved timing, as well as the shifted centres contracting the time separation between the kinematics critical points.

Figure 7f shows the distribution of trial time stamps. Each panel refers to one delta class -short or long. This distribution revealed an alternating strategy between the two trial classes. The number of trials that it took to switch from one class to the other across random reached was mostly 1 or 2, i.e. given trial n in one class, most likely trial n+1 would be in the other class. Despite this alternating strategy, when grouping trials per target, there was a slow monotonically increasing trend in each class that coincided with the order in which the trial was acquired. This is depicted in figure 7d for data from several consecutive days of training.

*The total time of the motion*: The movement duration relates to the total length of the trajectory so we measured both quantities and the other spatio-temporal parameters in each block. The OB-avoidance total path length ranged between 37 cm and 79 cm for ipsi-lateral targets and between 39 cm and 105 cm for contra-lateral targets. The partial distance delta travelled up to the first velocity peak varied between 10 cm and 36 cm for straight reaches and between 11 cm and 60 cm for curved reaches around 1 and 2 OB(s). The total movement duration time *t *in ms ranged between 420 ms and 1,600 ms for ipsi-lateral targets and between 500 ms and 1000 ms for contra-lateral targets. It was highly variable at the beginning of the learning, for example in a single session it evolved from 1,600 ms until it became stable at 700 ms in the most affected ipsi-lateral target.

Automated straight reaches showed consistent movement duration for each location despite the randomness of the target presentation. This contrasted with the earlier trials of the OB-avoidance learning block where the total duration was highly variable yet with a consistent monotonically decreasing trend at each randomly cued target location. The last portion of the OB-avoidance block was characterized by a more consistent duration in each target location. For each target location we observed statistically significant differences between the automated straight and OB-avoidance reaches. Likewise, there were significant differences within the OB-avoidance block when we compared earlier and later trials of the block, with a significant effect of target location (alpha level 0.01). Table 1 lists these results in detail for 6 of the most affected target locations.

### Invariant symmetries of the intended reaches

Despite significant differences in all trajectory parameters as a function of target location; and as a function of the learning stage of OB-avoidance, the area and the perimeter ratios pooled across all target locations and repeats for each animal formed a unimodal distribution clustered tightly around 1/2. This distribution was similar for both quantities and remained so despite striking differences in temporal dynamics (figure 4D) or hand path curvature (figure 5C).

The similarity of the area and the perimeter ratios denoting their co-variation was confirmed by the Friedman's test [27] yielding  for straight reaches, OB-avoidance with 1 and 2 obstacles; and during learning curved vs. automated curved reaches . Figure 4 shows the invariance of these symmetries and their co-variation for strikingly different temporal dynamics across space. Figure 5 shows their invariance to different geometries expressed in different families of hand trajectory curvatures. Furthermore a two-tailed pair wise t-test at the 0.01 alpha-level did not reject the null hypothesis of 1/2 mean-value for co-variation of the ratios, both during early learning and late automatic trials. Regression lines were obtained for each case and they yielded also significant similarity in slopes and intersections.

### Violation and recovery of the invariant symmetries and their co-variation in unintended reaches

**Figure 8:**
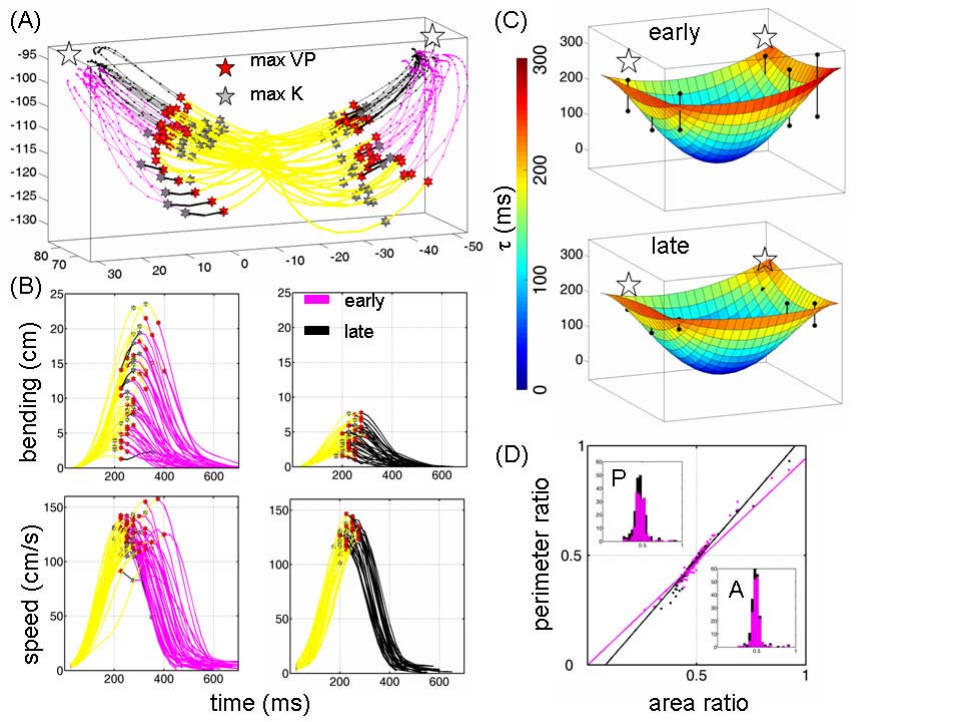
**Violation and recovery of the geometric invariant symmetry when there was a mismatch between intended and actual paths**. (A) Hand trajectories to targets in the absence of obstacles show the initial residual after-effects (magenta color) from the previous block when highly fast and curved automatic OB-avoidance motions had been executed to avoid obstacles. Such trajectory changes were not intended. The inertial forces still present in the arm seemingly overrode the intended abstract goals of reaching straight to the targets but eventually the performance converged to trajectories that were compliant with the goals (black) and the ratios were again 1/2. (b) Trajectory bending in the early (magenta) and late (black) trials of the same block and their corresponding speed profiles show the initial inconsistency of the (tau, delta) parameters which eventually became synchronized when the motion was once again automated. (C) The high variability of the spatial map of tau in the earlier trials contrasted with the stable consistent map in the late trials. The target locations in (A) are marked with stars on the map. (D) The invariant similarity of the area and the perimeter ratios broke down when tau was highly variable and there were unintended curved hand paths in place of a more desirable straight solution. Over the course of several trials the system recovered from this "perturbed" state and regained the invariant.

During the early trials of the third block of straight reaches (A') both subjects manifested motion trajectories similar to those observed during OB-avoidance. Their hand paths were curved and the temporal profiles along the hand paths were highly variable. A distinct difference was that the consistency of the parameter tau was no longer present. Eventually the simultaneity of the delta-tau pair quantified during the first pulse of automated straight reaches was regained in this block. Attaining this synchronicity however, took many trials (at least 5 per each of the 14 targets). At that stage we found as in the A-block, similar bimodal distribution of the percent of time to reach the maximum acceleration relative to the maximum speed, and a unimodal distribution of the distance delta travelled to reach the first velocity peak. Figure 8 shows the violation of the symmetries' co-variation, quantified by the failure of the Friedman's similarity test  for the earlier de-adaptation trials and the later automated straight trials.

## Discussion

This work investigated (1) if there were new movement regularities conserved across various families of goal-directed voluntary reaches; and (2) if the regular patterns across reaches would remain conserved despite high motion variability during the natural learning of a new procedural act. We found that during intended goal-directed reaches, despite significant statistical effects of target location and learning stage on many relevant spatio-temporal movement parameters, there was a new symmetry linked to hand trajectories. This symmetry was captured by two ratios of the hand trajectories, which significantly co-varied across space and remained invariant to changes in temporal dynamics, to changes in hand path curvature and to changes in the arm postural paths required to achieve differently curved hand trajectories across the three-dimensional physical space.

It is important to notice that all research questions addressed here are part of a research program investigating the cross roads between geometry and temporal dynamics during unconstrained intentional actions. This program aims at further developing the idea of an internal model of the body's temporal dynamics [28,29] by providing a representational model of the intended motion timing that can be compared to the actual timing from the movement's execution. Temporal dynamics relate to the total path length and to the speed-acceleration critical points of the motion (maxima and minima) defining key segments. Our new approach proposes that such dependencies on relative timings must play a role on how we plan and learn the temporal patterns of new and complex motions.

It is generally believed that an internal representation of movement variables -such as position and velocity- is maintained in the neural activity (whether in intrinsic joint and muscles or extrinsic coordinates) and used to update and monitor the state of the system. Such representations are thought to be stored as internal models [29-31] that may provide a neural representation of the body-environment interaction dynamics.

It is accepted that these internal models can be characterized by dependence on a motion state -a mapping between motor commands and resulting limb motions-rather than by a dependence on the times ***when ***they occur [32]. Empirical support for this general assumption is limited to a few force field-adaptation and learning studies specifically addressing the absolute timing issue [33,34]. No evidence for a central absolute representation of time -a ticking clock dictating the moment-to-moment timing of state variables-has ever been found. Interestingly, no previous studies have addressed the relative timings between acceleration and velocity state variables, even though the learning of different force patterns may require the coordination of such relative timings in order to control motor learning at different time scales.

The present work uncovered a bimodal distribution of maximum acceleration-magnitude relative to maximum velocity-magnitude suggesting that relative timings are indeed important to learn new curved point-to-point reaches. Each bump of the unveiled bimodal distribution reflected separable acceleration timing relative to the maximum speed, suggesting that -generally for both automated straight and automated curved timings-the peak velocity can serve as a critical reference point.

These data demonstrate that in addition to a dependency on the motions' states, the system's performance in general straight and curved reaches also manifests a dependency on the times *when *these states occur. In particular the relative timings of these states' critical points (maximal acceleration relative to maximal speed) appear to play an important role in the learning of new curved reaches. Models of motor learning that treat time as a free parameter and that leave out such relative-temporal dependencies will not account for these empirical results of unconstrained three-dimensional complex reaches performed at the subjects' own pace.

In both automated straight and curved OB-avoidance reaches studied here the hand path length remained consistent despite the temporal learning and despite the arm's redundant d.o.f. Motivated by these empirical results, in all 3 cases (straight, 1OB and 2 OB(s)) we examined the portion of the total path length that it took the hand to reach maximum velocity (denoted delta in cm) and that lasted tau ms. These parameters indicate ***where ***and ***when ***in the path the hand attains the speed maximum. The temporal analyses had revealed the speed maximum as a possible critical point of reference to time the reach. The spatial analyses revealed that these critical points largely depended on the reliable path length -used to update relative distances during learning.

The questions addressed in this work were motivated by our geometric characterization of intentional arm-hand motions. In each case we had previously characterized the curve to reach a target as the shortest-distance path with respect to a task-dependent Riemannian distance metric. In curved reaches for the (delta, tau)-associations, we had proposed that these parameters could play a key interchangeable role in predicting ahead sensory-motor temporal lags [9]. We systematically found here that tau and delta did play important roles in the relative timings of acceleration and speed during automated reaches. During the learning stages of new curved timings, the time and distance parameters inverted their roles as the system converged to an (automated) synchronous first pulse.

The hand path curvature played a significant role on this motor-learning process as it determined the cases in which the learning of the new motion timing was based on the adjustments of the distances travelled by the hand at a fixed time-length in relation to the maximum speed. In this regard it was important to divide the hand trajectory into different critical temporal land marks defining different segments along the motion path. This further relative-timing refinement revealed that later points along the temporal path were more affected by the error-correction process. This process was driven by the trial-by-trial adjustments of the distance travelled by the hand after the maximum speed had been attained, prior to reaching the intended target. Eventually the hand converged to a smooth timing and travelled two segments. The overall motion then consistently lasted a similar time length.

***We speculate ***that it is possible that during the later portions of the movement trajectory sensory-motor feedback was used to correct errors between intended and actual executed dynamics. This was evidenced in the later corrections of the jerky speed profiles, which disappeared as the earlier delta became consistent. In contrast to the later corrections, in the earliest pulse of the trajectory (so-called open loop) information on the distance-to-be-travelled seemed more adequate than actual motor feedback (possibly not yet fully available) to estimate adjustments in the upcoming dynamics and to achieve (eventually) a systematically consistent overall timing. In this earlier portion of the reach the system may have placed a tighter bound on the variability of the length of time devoted to the segment. This was evidenced in the low variability of the parameter tau throughout the learning process.

Regarding the interactions between geometry and relative-timing, the rate of change of the distance accumulated along the highly curved hand path in principle interacts with the acceleration of the curve at each point (the vector perpendicular to the velocity vector tangent to the curve). If there had been extreme changes in distance travelled, the hand would have fallen out of the intended direction along the path and collided with the OB(s). Yet this was not the case during curved-timing learning. Throughout the learning process -despite different dynamics - the curvature of the path and its overall length were systematically conserved across random repeats to the same target.

Path length conservation facilitated distance-based adjustments when learning the curved timing. The unveiled bimodal distribution of delta manifested during the adjustment phase contained trials from all targets. This uniformity ruled out an explanation of the non-unimodal distribution solely based on the subjects' biases due to comfort (preferred spatial target location) or effort (fatigue in the later trials of the block). The composition of the classes showed mostly an alternating distance-adjusting strategy where if trial n was in the short-length trial class then trial n+1 would most probably be in the long-length trial class. This systematisation suggested that the system was running two learning processes simultaneously, one short-term based on trial-by-trial error corrections and one long-term quantified across days of training. We had previously tracked longitudinally the long term learning ([9] and see also figure 7g), but the short-term process presently addressed here revealed new general relative-timing features of both straight and curved reaches (figure 7c).

The present results fully agree with previous reports, which had suggested different time scales for motor learning in the context of a different force-field paradigm [18]. In the force-field context, motor learning is studied under high spatio-temporal constraints and external forces which were not imposed in the present work. Our results extend the previous findings to motor learning in three dimensions, recruiting more of the arm's d.o.f. and occurring at the subjects' self-determined, preferred tempo. This suggests that motor learning at different time scales seems to be a general feature of the primate arm system, manifested whether or not movement time is bounded a-priori by the experimenter.

The data from both animals showed that whenever the intended path geometry and tau were consistent, the symmetries and their co-variation held. We found that the uncovered invariant of hand motions was violated whenever the executed motion path miss-matched the intended path. This was the case during the de-adaptation trials where different forces were still present in the arm system from the previous experimental block. To describe this new phenomena we borrowed the term "after-effects" from the well known motor learning paradigm that uses force fields to alter arm dynamics [35]. In our case however, unlike in the force-field paradigm, no external perturbations had been imposed to the arm. The observed effects were endogenous in origin and came as a residual force from the previous OB-block, where highly curved and fast trajectories had been repeatedly executed. The system was "perturbed" out of its default state and this perturbation inevitably over ruled the intended path straight to the target. It took many repeats for the system to de-adapt from the highly fast and curved motions and to recover the invariant. After several trials (approximately 70, 5 per target) the invariant symmetry and the co-variation of the ratios was again expressed in the hand motion trajectories. The intended straight paths defined by the goal once again matched the actual executed paths.

During the de-adaptation trials tau was highly variable at each target location. This was in contrast to the other blocks of automated straight reaches, and OB-avoidance learning where this temporal landmark of the hand trajectory remained consistent. In those cases, we had found that the variability of tau was negligible compared to the variability of delta. We had quantified the systematic consistency of tau even during the striking changes in overall temporal dynamics from motor learning, yet during de-adaptation tau varied significantly between the mean values of the 2 motor programs for straight and curved paths.

During de-adaptation trials the parameter delta also had comparable variability to that of the parameter tau. An immediate sensory-motor strategy was not evident in these trials at first. The inevitable arm state seemed to have over ruled the intended sensory-motor transformation strategy. Yet eventually, after many trial-and-error repeats, with no apparent systematic pattern of variability in the hand trajectories, the system regained synchrony of delta and tau, and the bimodal trial-distribution of α was again quantified. Perhaps systematic de-adaptation patterns could have been identified in the arm muscles domain, but our recordings of the arm kinematics at 120 Hz limited our ability to address different sources of noise and their possible meanings with regards to important lower-level control strategies.

The invariance of the uncovered symmetry may reveal a higher-level sensory-motor transformation and integration strategy common to several families of reaches performed by the primate arm system. The learning progression from the straight to the curved timings revealed important features. The non-unimodal distribution of α in the automated straight reaches disappeared when learning the curved timing. As the system built a new curved temporal profile, the α-distribution turned unimodal and the δ-distribution turned non-unimodal. Upon examination of relative timings at the end of the learning process, we discovered that the newly automated curved trajectories had similar non-unimodal relative-timings structure as the automated straight trajectories. The spread of the clusters had however changed and the centres shifted. In the new curved timing there was a contraction of the time-length separation between the speed and the acceleration maxima.

The OB-avoidance learning strategy was closely tied to the rate of change of the distance accumulated along the first pulse of the reach in relation to the overall path length: i.e. the percentage of the path covered. The path length remained stable (low variability) throughout the curved-timing learning process both in the postural and in the hand domains. This stability in the face of so many possible postural configurations suggested an initial geometric strategy based on relative distances rather than on relative timings. At the end of the learning process however (figure 7c), the straight relative-timing profile had transferred to the curved path.

*We speculate *here that in visuo-motor acts the learning of complex curves in high dimensions and their projection to three-dimensional space may initially rely on sensing the distances covered by the end effector (proprioceptive sense of posture) and estimating ahead (through vision or a visually-guided memory) the remaining distance for various segments. Eventually, through trial and error performance, aiming for a faster, less time-segmented motion, the system may learn to rely on the relative timings, as was manifested in the transfer from automated straight to automated curved reaches. There is evidence that smoothness maximization is important to the motor system [4,36], yet relative timings -which we found here for more complex curved three-dimensional motions-had never entered as a goal in earlier characterizations of the motor-control planning problem.

In our previous work [9] the longitudinal study of these parameters -from when the animals were naïve to when they were highly trained and proficient-had revealed a decoupling of the spatio-temporal parameters and pointed to the distance-related parameter as a key element for the learning of the motion's new tempo. The present study confirms this observation and provides further evidence that the system can use spatial distance-sensing in the dark to guide the learning of new curved timing.

### Geometric Interpretation

We have previously proposed [9,11] that during motor skill acquisition the motion curves described by the arm-hand system can be characterized using Maupertuis'-Jacobi's "Principle of Least Action" from variational mechanics (Feynman 1965; Lanczos 1970; Jose and Saletan 1998). This principle considers mechanical systems whose Lagrangian function does not contain time explicitly, and brings out the relationship between conservative systems and the non-Euclidean geometry of the underlying configuration space. The "Principle of Least Action" establishes that the problem of finding the solution of a given dynamical problem is mathematically equivalent to the problem of finding the geodesics of the underlying space. In particular the description of the motion paths in the learning stage can be computed as the shortest "straight line" (a geodesic) between two definite end-points in a Riemannian manifold [9,11] independent of the time of the physical motion. According to our model, the invariants described here emerge from the correct spatio-temporal alignment between internally- and externally-based sensory input time-lags to preserve the map/transformation between the intended action curves and the corresponding actual execution's dynamics. This conservation is independent of the specific metrics or coordinate functions of choice, but sensitive to the isometric features of the map(s) that define the relations between points in the sensory-motor spaces of interest.

According to our geometric model, under normal conditions, the learning of new dynamics along new curved paths would entail building an association between the general notion of distance (in a sensory space) to be travelled along a geodesic direction (pointing along the shortest-distance path) and the time lag that it would take for sensory-motor feedback to return and be utilized to complete the segment. Once the motor program became automated, the time lag (which we sense from movement) and the distance (which we can mentally represent in some sensory space) would be interchangeable, so we could have a representational model of the dynamics in a "Least-Action-Principle" sense; i.e. we could have a representational, time-invariant geometric model of the dynamics motion path. To the naïve system distance and time would be separable but to the automated system they would be equivalent. Whenever this association-map became ambiguous or misaligned (as during de-adaptation or brain injury [23]), such correspondence would be violated and so would be the invariant symmetries and their co-variation. In this sense this is an invariant that pertains to motions that are compliant with intended sensory-motor transformations.

## Conclusion

We have previously found in similar ***memory-guided reaching ***contexts that adult human Parkinson's and Parietal patients also violated this invariant, yet they could repair the symmetries and their co-variation when the appropriate source of sensory guidance was provided to cue the spared systems. The deficit/repair pattern of the symmetry was specific to the injured site and so was the preferred source of sensory guidance. The present work complements the patient data and strongly suggests that this movement regularity is not merely a by-product of the primate arm's biomechanics and its compliance with the physical laws of motion. We believe that this invariant reflects compliance with intended sensory-motor transformation strategies of the primate brain and conclude that it is a manifestation of mental laws governing the control of voluntary arm movements.

## Competing interests

The author declares that they have no competing interests.

## Authors' contributions

All authors read and approved the final manuscript. EBT designed the task, programmed the motion capture recording system, designed the questions addressed in this paper, designed the analyses and modeled the phenomena prior to the experiments. EBT trained the subjects, carried on the experiments, collected and analyzed the data and wrote the paper. EBT did not build the experimental set up which was part of the Andersen laboratory at CALTECH. In addition to this, as part of a broader question, Professor Richard Andersen posed the question of curved trajectories in relation to the Posterior Parietal Cortex and provided the idea of using obstacles to evoke hand path curvature.

## Appendix 1

**Figure 9:**
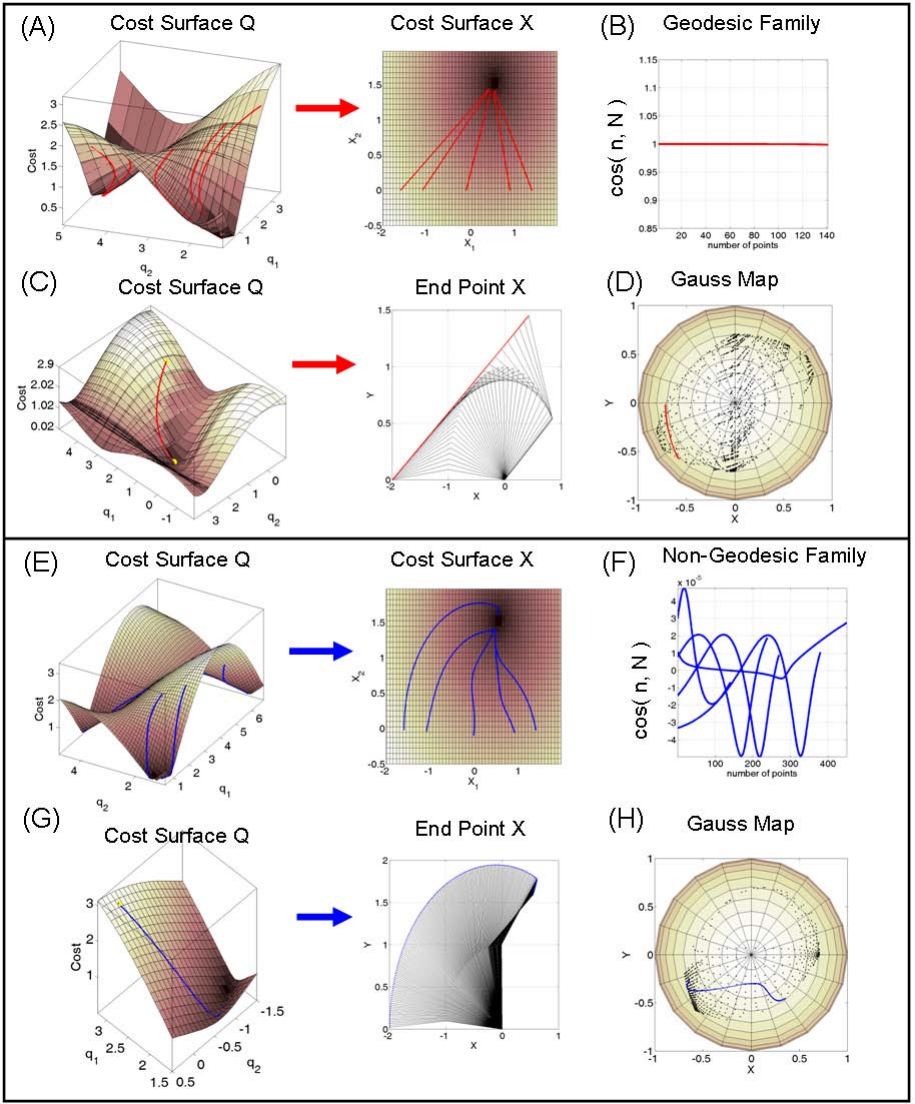
**Geodesic vs. non-geodesic families of sensory-motor path transformations (see appendix 1)**.

Paths generated with the gradient-flow-driven equation *dq *= -*G*^-1^∇*r*° *f*(*x*^target^, *q*^init^)Δτ (i) described in [11] conserve their length-minimizing property under local isometric embedding (transformation). The Gauss Map of the vector  normal to the gradient flow at each point on the Cost surface locally preserves the geodesic property (see Additional file 1 for terminology). The gradient-path projected on the unit sphere is on a great circle (Figure 9A-D). In such cases the projection of this path to the Euclidean three-dimensional straight line and the computation of the trajectories' area-perimeter ratios reveal a symmetry and similarity. Whenever this transformation is not distance-metric preserving the ratios will not be 1/2 and they will not co-vary (Figure 9E-H).

Figure 9(A) shows a geodesic family of postural trajectories on the Q-Cost surface whose coordinates are q_1_-shoulder, and q_2_-elbow rotations. Parameterization of the surface is compatible with the metric and projects geodesics paths on the Q-Cost to geodesics paths on the X-Cost surface according to equation (i) (fully described in [9,11]) which measures the Cost (distance) along the z-axis and descends to the target location *x*^target ^pulled back into the corresponding *q*^target ^in Q according to the gradient rule. This gradient flow minimizes the remaining (non-Euclidean) distance between current posture in Q and final target in X subject to any additional priorities and constraints that the task at hand may demand [9,11]. This geometric construction preserves the shortest distance path property and transforms "straight-lines" from one space to the other. This is achieved with a change of metric due to a change of coordinates when pulling back from X to Q.

In the toy-model example of Figure 9 the forward map is given by ,  representing the arm's endpoint.

The metric in X is pulled back into  and

the metric in X is 

The cost function in this case is 

In Figure 9(B) the gradient parallels the principal eigenvector of the Hessian (associated to the Second Fundamental Form [37], see Additional file 1) ∇^2^*r*° *f *along the path. This is measured by  indicating that the normal  to the surface at each point along the path parallels the normal  to the gradient vector, which is the acceleration of the curve. This means that the geodesic curvature is 0 (see Additional file 1). The normal to the gradient does not pull away from the normal to the surface at each point along the path. The gradient flow parallel-transports the normal vector to the surface (and to the gradient) from the start to the end of the simulated reach. All points from all 5 paths fall on the line of .

Figure 9(C) focuses on a single path from the family and its projection to the Euclidean straight line on the Cartesian plane. Notice that even in 2 dimensions with no joint-angle redundancy finding the path is an ill-posed problem as different paths in Q correspond to the same path and end point target in X. In Figure 9(D) the Gauss Map projects the geodesic path in Q to a great circle on the unit sphere and conserves the geodesic property because the gradient equation builds in general a local isometric embedding when the metric and parameterizations are compatible as in this case. The projection of this great-circle path on the Euclidean straight line in three dimensions yields the symmetric area-perimeter ratios and they co-vary in such ideal cases where the local embedding is isometric. The black dots are the projection on the unit sphere of , the vector normal to the surface at each point. Figure 9(D) shows the same family as in (A) in the case when the metric and Q-parameterization are incompatible and the family of paths generated with the gradient equation are non-geodesics (are not length-minimizing curves). The projection on the X-Cost surface has excess curvature. The transformation between Q and X does not preserve the length minimizing property. In Figure 9(F) the gradient and the leading eigenvector of the Hessian are no longer parallel, so their dot product (the cosine of their angle) is no longer 1. Figure 9(G) focuses on a single postural path on the Q-Cost surface. This q-path projects to a curved x-path that does not minimize the notion of Euclidean distance. Figure 9(H) shows that the path on the Gauss Map does not fall on a great circle of the unit sphere. In this case the transformation map does not preserve the geodesics property. The projection of these kinds of paths on the Euclidean straight line neither yields the symmetric area-perimeter ratios nor their co-variation.

The results illustrated here in lower dimensions extend to higher dimensions for a more realistic characterization of this problem Figure 6.

Figure 6(A) shows the many-to-one feature of the *f*-map from postures to hand position. The star marks an abducted posture used later in (D) to simulate a reach. Figure 6(B) shows the case of reaching a target from a comfortable posture and travelling along a length-minimizing path. Figure 6(C) shows the path deformation through a change in distance metric and an isometric transformation from hand to posture space. The same target location was used in the simulation of this change in geometry. The cost included two main segments in this case, one to pull the hand to travel a given delta, it is turned to 0 as the hand attains that critical point and the other term takes over to complete the second motion segment. The cost in this case was defined by 2 δ-segments but in general for more complex motions one needs to define several δ-segments:  driven by the , where D_i _is given as a visually sensed desired distance quantity and δ_i _is the distance traversed that changes as the gradient flow changes the hand position, (*) is the sign which alternates as each segment is completed to drive the term towards 0 [9].

The distance-based formulation presented here can be converted to a time-based formulation of relative-temporal segments tracking differences between angular and linear velocities and accelerations. In addition the λ-terms can be defined by other smooth-differentiable real-valued functions to represent different tasks. Since the gradient is a linear operator, the gradient of the sum (of all segments adding up to the total length or duration of the reach) equals the sum of the individual gradients of each segment. Thus the segmented learning process can be modelled using the general gradient equation (i) and the excess segment-terms that make the motion jerky can be turned to 0 as the system learns to smooth out the timing. Likewise, more complex realistic multi-segmented motions in posture space can be geometrically characterized using the gradient approach. In such cases the limb segments can be synchronized or de-synchronized using a higher level temporal coordination scheme. Figures 6(D)-(E) show the results of simulating the motion along a length minimizing path from different initial postures to reach the same target. Figure 6(F) shows the three-dimensional hand traces from gradient flows that preserve the symmetries and their similarity in different geometries.

Notice that this general gradient-flow solution could be applied to other non-brain problems. Thus, it is not implied here that this is the brain solution to this set of problems. What seemed striking were two findings: (1) this arbitrary model predicted area-perimeter related symmetries that held in actual *primate* arm motions of several kinds (it would have not been possible to have a-priori constructed this result in a series of reaching experiments, or under present motor-control schemes to have a-priori controlled the motions of a robotic arm in such a way); (2) the simulated situations in which these symmetries and their co-variation would break down also existed in the empirical case. In the model this violation was predicted for non-isometric mapping of the gradient flow. In the after-effect data the invariants were indeed violated when the system mismatched the intended straight and the actual curved motion-path flow. The real system however, eventually recovered the symmetries and their similarity thus suggesting a default preference for a sensory-motor transformation strategy. This strategy seemed congruent with what the model had specifically predicted under local isometric embedding.

This suggests that under normal circumstances the geometric transformations that give rise to actual motion paths from the primate arm can be on average well characterized by the local isometric embedding of the proposed PDE. Furthermore despite motor noise the symmetries from the data manifested invariance to straight or curved timings. The actual trajectories could be on average well characterized by different families of length minimizing curves and still preserve the similarity of the time-invariant symmetric ratios so long as -regardless of the sensory space geometry-the transformation from visual to body coordinates was distance-preserving (isometric).

Both in the model and in the veridical paths the ratios were shown to be independent of the curvature and timing. Previous empirical data had shown the speed invariance of voluntary postural and hand reaching paths [9,10,38,39] even when loads were attached to the arm. Because this measure is insensitive to changes in temporal dynamics and emerges from coordinate transformations similar to those which the brain requires to perform in sensory-motor integration processes, we can use it as a model to monitor the departure of the primate arm system's motion paths from the geometrically "ideal" cases. Further theoretical work will be necessary to fully understand the new empirically unveiled straight-curved time-distance equivalence.

## Supplementary Material

Additional file 1 Supplementary material contains a glossary of geometric terminology and a proposed computational method to further pursuit the study of this invariant symmetry in more complex motions. Click here for file
