# Supplementary material for: New symmetry of intended curved reaches
Source: Behav Brain Funct. 2010 Apr 1;6:21. doi: 10.1186/1744-9081-6-21 (PMC2867946; doi:10.1186/1744-9081-6-21)
Supplement: Additional file 1 — Supplementary material contains a glossary of geometric terminology and a proposed computational method to further pursuit the study of this invariant symmetry in more complex motions. [file 1744-9081-6-21-S1.DOC]

1. Supplementary Material: Definitions and Terminology

The **line element** $\overline{ds}$is the quantity denoting the infinitesimal distance between two neighboring points of space, expressed in terms of the coordinates and their differentials. In the Pythagorean case, $\overline{ds}^{2}=dx_{1}^{2}+dx_{2}^{2}+\ldots$. The expression is a consequence of the Euclidean postulates and the coordinates $x_{1},x_{2},\ldots$However, if the coordinate lines of the reference system are no longer straight lines, but arbitrary curves, then the following general form is used instead (1):

$$\overline{ds}^{2}=\sum_{i,j=1}^{n} g_{ij}dx_{i}dx_{j}$$

The quantities$g_{ij}$’s are the coefficients of the special metric tensor, also known as the **First Fundamental Form**. They are the elements of a symmetric, positive, definite matrix, which in flat space is the identity matrix. In general, they are constants only if rectangular, or more generally, “rectilinear” coordinates (with oblique instead of rectangular axes) are used. In the case of curvilinear coordinates, the$g_{ij}$’s change smoothly from point to point.

In our model of the arm let $X^{m}$and $Q^{n}$be differentiable manifolds,$m<<n$. A differentiable mapping $\phi:X\to Q$ is an **immersion** if $d\phi_{p}:T_{p}X\to T_{\phi\left(p\right)}Q$ is injective$\forall p\in X$. The difference $n-m$ is the **codimension** of the immersion.

If $\phi$ is a continuous one-to-one mapping with a continuous inverse, (a homeomorphism) onto its image$\phi\left(X\right)\subset Q$, $\phi$ is an **embedding**. If$X\subset Q$and the inclusion is an embedding, then *X* is a **submanifold** of *Q*.

A function$f:Q\to X$is an **isometric mapping** between metric spaces$\left(X,d_{X}\right)$ and $\left(Q,d_{Q}\right)$ if and only if *f* preserves distances:$d_{X}\left(x,y\right)=d_{Q}\left(f\left(x\right),f\left(y\right)\right)$ $\forall x,y\in X$. Two metric spaces are isometric if there is an isometric mapping from one to the other.

A distance-preserving embedding is an **isometric embedding** (1).

Let$S\subset R^{3}$be a surface with an orientation *N*. The map$N:S\to S^{2}\subset R^{3}$that takes its value on the unit sphere:$S^{2}=\left\{\left(x,y,z\right)\in R^{3};|x^{2}+y^{2}+z^{2}=1\right\}$is called the **Gauss Map** of *S*. The differential of this map operates on the tangent spaces to the surface and the sphere respectively:$dN_{p}:T_{p}S\to T_{N\left(p\right)}S^{2}$. But it is known that in$S^{2}$ the tangent plane at the point is normal to the position vector; i.e. tangential to the original surface, so$T_{p}S||T_{N\left(p\right)}S^{2}$, and we can express its differential as acting on the tangent space, $dN_{p}:T_{p}S\to T_{p}S$ (**SM Figure 1**).
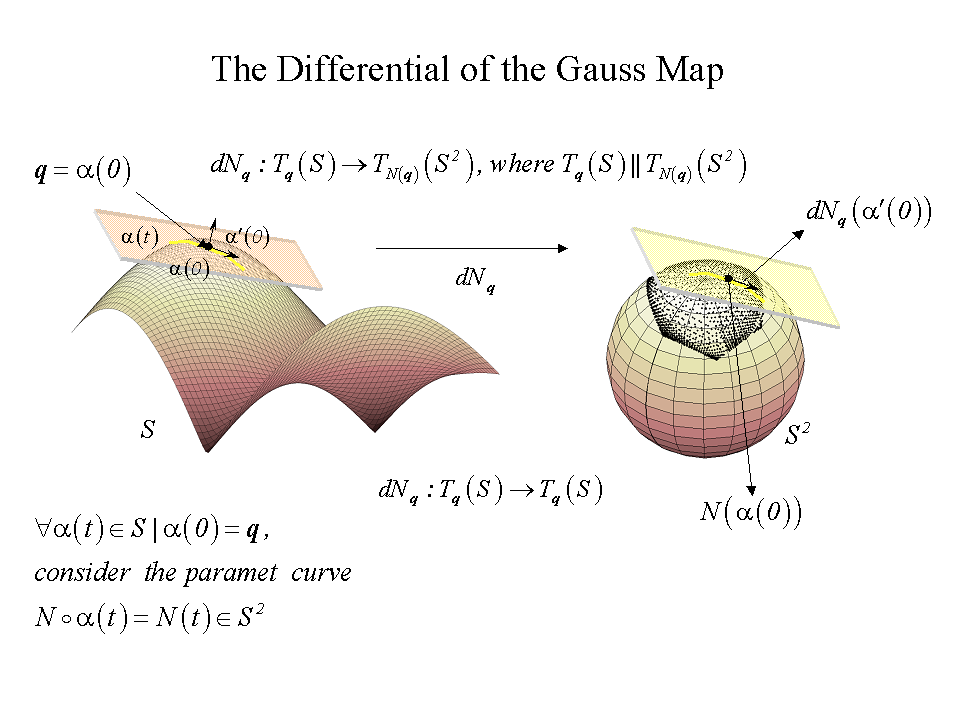


***SM Figure 1 Illustration of the differential of the Gauss Map using the gradient-flow model. Curves on the surface S and unit sphere S2 were generated with the gradient equation (See Appendix).***

The$dN_{p}$ is a linear, self-adjoint map associated to a quadratic form that acts on the tangent space *(2,* 3),$Q\left(v\right)=-\left\langle dN_{p}\left(v\right),v\right\rangle,\text{ }v\in T_{p}S$, also known as the **Second Fundamental Form** of *S* at ***p***:$II_{p}\left(v\right)=Q\left(v\right)$ with the following geometric interpretation:

Consider the regular curve *C* on *S* passing through$p\in S$, *C* parameterized by$\alpha\left(s\right)$, where *s* is the arc length. The curvature of *C* at ***p*** is *k*, and$\cos\left(\theta\right)=\left\langle\vec{n},\vec{N}\right\rangle$, where $\vec{n}$ is the normal vector to *C* at ***p***, and$\vec{N}$is the normal to *S* at ***p***. The number $k_{n}=k\cos\left(\theta\right)$ is **the normal curvature** of *C* in *S* at ***p***.

For a vector$\vec{v}\in T_{p}S$, $II_{p}\left(\vec{v}\right)$measures the normal curvature of the curve passing through ***p*** and tangent to$\vec{v}$(**SM Figure 2**). In our formulation ***p = q*** a posture and $\vec{v}=dq$ defined by the gradient.


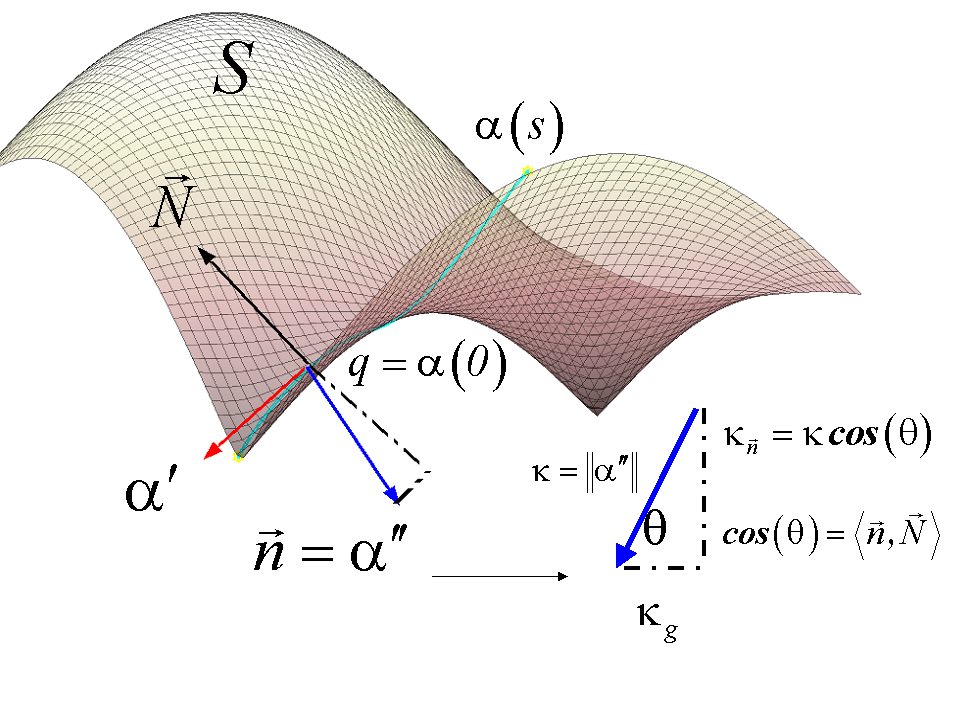


***SM Figure 2 Illustration of the geodesic and normal curvatures.***

To see this, let $p=\alpha\left(0\right)$ and $N\left(s\right)$ be the restriction of the normal vector to the curve$\alpha\left(s\right)$. The vector tangent to the curve at the point, $\alpha'\left(s\right)$ is orthogonal to the restriction$N\left(s\right)$: $\left\langle N\left(s\right),\alpha'\left(s\right)\right\rangle=0$and$\left\langle N\left(s\right),\alpha''\left(s\right)\right\rangle=-\left\langle N'\left(s\right),\alpha'\left(s\right)\right\rangle$, so:

$$\begin{matrix} II_{p}\left(\alpha'\left(0\right)\right)=-\left\langle dN_{p}\left(\alpha'\left(0\right)\right),\alpha'\left(0\right)\right\rangle=-\left\langle N'\left(0\right),\alpha'\left(0\right)\right\rangle\\ \text{ }=\left\langle N\left(0\right),\alpha''\left(0\right)\right\rangle=\left\langle N,k\cdot n\right\rangle_{\left(p\right)} \\ \text{ }=k_{n}\left(p\right) \end{matrix}$$

The acceleration$\alpha''\left(s\right)=\alpha''\left(s\right)^{T}+\alpha''\left(s\right)^{\perp}$ has a tangent and a normal component and by Pythagoras$\left\|\alpha''\left(s\right)\right\|^{2}=\left\|\alpha''\left(s\right)^{T}\right\|^{2}+\left\|\alpha''\left(s\right)^{\perp}\right\|^{2}$can be written in terms of the geodesic and the normal curvatures,$\left\|k\right\|^{2}=\left\|k_{g}\right\|^{2}+\left\|k_{n}\right\|^{2}$.

When for each point in the curve,$\cos\left(\theta\right)=\left\langle n,N\right\rangle=1$, i.e. the normal to the surface$\vec{N}$is parallel to the normal$\vec{n}$to the curve at the point, the path is a **geodesic**. In other words, the acceleration is perpendicular to the normal$\vec{N}$and the geodesic curvature is 0.

These notions generalize to higher dimensions, so we can apply them to study the geometry of surfaces related to problems involving$n>3$dimensions: Given a differentiable function$r\left(p\right)$, we can always study the surface *S* corresponding to its graph, where points are of the form$\left(p_{1},p_{2},\ldots p_{n},r\left(p\right)\right)$.

One can define the unit-normal vector field on the surface *S*. Each$\vec{N}$is the unique unit vector at each$p\in S$orthogonal to$T_{p}S\subset T_{p}R^{n}$and chosen so that$E_{1},\ldots,E_{n},\vec{N}$ form a frame at ***p*** with the same orientation as the standard orthogonal frame $\left(\frac{\partial}{\partial x^{1}},\ldots,\frac{\partial}{\partial x^{n+1}}\right)$in$R^{n+1}$. Length and orthogonality are defined in terms of the inner product of Euclidean space, which induces a Riemannian metric on *S* by restriction. The shape of *S* at$p\in S$can be studied by means of the derivative of$\vec{N}$in various directions tangent to *S* at ***p***.

In particular, we are interested in the case where$p\left(t\right)$is a differentiable curve on *S* parameterized by arc length, with$p\left(0\right)=p$andin the tangent space. Restricting$\vec{N}$to$p\left(t\right)$gives a vector field$N\left(t\right)=N_{p\left(t\right)}$along$p\left(t\right)$which may be differentiated in$R^{n+1}$as a vector field along the space curve, giving a derivative which is itself a vector field along$p\left(t\right)$:

$\frac{d}{dt}\left\langle\vec{N},\vec{N}\right\rangle=\left\langle\frac{d\vec{N}}{dt},\vec{N}\right\rangle+\left\langle\vec{N},\frac{d\vec{N}}{dt}\right\rangle=2\left\langle\frac{d\vec{N}}{dt},\vec{N}\right\rangle=0$.

Using$\left\langle\vec{N},\vec{N}\right\rangle=1$, we have that$2\left\langle\frac{d\vec{N}}{dt},\vec{N}\right\rangle=0$, which means that$\frac{d\vec{N}}{dt}$ is orthogonal to$\vec{N}$at each point$p\left(t\right)$and hence tangent to *S*,$\therefore\frac{dN}{dt}\in T_{p}S$. We restrict our attention to$p\in S$, and consider a curve through it with $p\left(0\right)=p$and tangent vector. The vector $\left(\frac{d\vec{N}}{dt}\right)_{s=0}$depends only on$X_{p}\in T_{p}S$, and not on the curve chosen. Let $\Phi\left(X_{p}\right)=-\left(\frac{d\vec{N}}{dt}\right)_{t=0}$then$X_{p}\to\Phi\left(X_{p}\right)$is a linear map$\Phi:T_{p}S\to T_{p}S$(as the Gauss Map for surfaces imbedded in$R^{3}$described earlier). This map can be determined at each point in *S* to define a covariant $C^{\infty}$tensor field on *S* (assuming *S* is a $C^{\infty}$submanifold).

Let $\Phi:V\to V$be a linear operator on a vector space *V* with inner product$\left\langle X,Y\right\rangle$, then the formula$\Psi\left(X,Y\right)=\left\langle\Phi\left(X\right),Y\right\rangle$defines a symmetric-bilinear covariant tensor of order 2 on *V*,$\Psi:T_{p}S\times T_{p}S\to R$is the **Second Fundamental Form**$II_{p}\left(X,Y\right)$described earlier. The quadratic form associated to it for a vector$X_{p}\in T_{p}S$tangent to the curve is $Q\left(X_{p}\right)=\Psi\left(X_{p},X_{p}\right)=\left\langle\Phi\left(X_{p}\right),X_{p}\right\rangle$ *(2,* 3).

In the case of interest, where the surface$S=\left\{\left(q_{1},q_{2},\ldots,q_{n}\right)\in R^{n};q_{n}=r\left(q\right)\right\}$,$\Phi$is the Hessian of$r\left(p\right)$. The eigenvectors of the Hessian together with the normal $\vec{N}$form an orthonormal coordinate frame useful to study curvature along the surface. In particular, the gradient vector defined by our equation$X_{p}=\nabla r$ tangent to the curve at ***p*** is the principal eigenvector of the Hessian, and the corresponding eigenvalue $\lambda=Q\left(X_{p}\right)=k\cos\left(\theta\right)$ (the normal curvature) is an extremal of *Q* over all unit vectors$X_{p}\in T_{p}S$. Our model of reaching tasks illustrates the property of the proposed gradient formula which generates geodesics paths on S, i.e. the geodesic curvature is 0 at each point in the path, the gradient vector is the leading eigenvector of the Hessian $\nabla^{2}r\circ f\left(x^{target},q^{init}\right)$and the corresponding eigenvalue is the normal curvature.

2. Proposed Computational Methods to Further explore the invariant

In previous work *(4*-6) we had characterized the reaching paths of several pointing, orientation-matching and obstacle avoidance motions using a partial differential equation (PDE) as a geometric solution to the problem of finding a unique solution path in the face of redundancy due the dimensional disparities of internal and external sensory-guidance spaces:

$dq=G_{\mu}^{-1}\nabla r\circ f\left(q,x^{goals}\right)\Delta\tau$ (i)

The solution curves to this PDE are geodesics (length minimizing curves) with respect to the G-metric used to both define the task and to express the initial conditions, the goals and the constraints of two spaces of disparate dimensions. These may include target location, target orientation, obstacles along the way, postural rotation bounds, etc.

In equation (i) $G_{nxn}^{\mu}=J_{mxn}^{T}G_{mxm}^{\alpha}J_{mxn}$ (ii) represents a change of metric due to the change in parameters expressed in the Jacobian transformation matrix, with dimensions m<<n.

The gradient solution can be generally applied to various problems domains in science and engineering where two spaces of disparate dimensions are required to represent the phenomena under study. One flows down the path that minimizes the remaining distance to the final goal according to the distance metric that the situation in question defines. The PDE defines an isometric transformation that preserves the geodesic property of the curves generated by the gradient flow.

In the case of the arm system m represents the dimensions of the task (e.g. 5 when matching an externally defined orientation to an internally defined posture as to conserve effort and energy (5), 4 in orientation matching without constraining the arm postures (6), 3 in pointing with or without obstacles (4), etc. The n represent the number of rotational joints in the arm, e.g. 7 in the model. The gradient-solution paths of (i) flow in both spaces and converge to the final goal in three-dimensional space, the corresponding arm configuration in posture space, while complying with additional conditions that the task may define in both spaces.

The model has been described elsewhere and used successfully to characterize different real experimental tasks *(4*-7) in humans and monkeys. The idea is to have an “ideal” curve to measure the departure from it in the veridical data. Here we focus on the deformation of these paths using variational principles to smoothly transition from one family of geodesics to another in order to systematically explore the area and perimeter ratios defined in the paper across different families of length minimizing curves from different geometries. We question to what extent the characterization of these empirical paths as length minimizing curves using (i) could explain the empirically described ratios and their unveiled relationships when we deform the metric but preserve it under transformation of coordinates. The brain faces a similar problem as it has to transform from one set of coordinates to another under different task conditions, so it is important to understand (1) if geometric relations in one space transfer to another and (2) under what conditions they do not.

**First**, to characterize the empirically obtained curves as length minimizing curves with equation (i), we define the metric in three-dimensional space and its pullback in seven-dimensional joint angle velocity space (the tangent space to the posture manifold) that can best fit the experimental curves. To this end we combine an analytical expression for the distance metric and numerically approximate the $g_{ij}$ coefficients that the analytical expression cannot account for (4)-Appendix.

This is achieved with a linear transformation matrix conditioned to be symmetric positive-definite in joint angle space. This procedure amounts to a point transformation of the joint angle parameterization to obtain the gradient of the cost *r* and flow accordingly *(4,* 5); or it can also be understood as a change of metric under the old joint angle parameterization. In either case we follow the gradient flow of $r\circ f$ to generate paths with 0 geodesic curvatures, i.e. curves where the normal to the cost surface parallels the acceleration vector to the curve (perpendicular to the unit gradient vector).

We refer the reader to the terminology above. The geodesic property measured by the Second Fundamental Form (SFF) of the Cost surface S, $\Pi_{q}\left(S\right)=\left\langle N\left(q\right),ddq\right\rangle=-\left\langle dN\left(dq\right),dq\right\rangle$ is depicted in **Appendix Figure 8** and illustrated for planar-arm cost surfaces. The SFF gives the rate of change of the normal$\vec{N}$to the Cost surface as the acceleration vector normal to the gradient *dq*, $\vec{n}=ddq$ pulls away from it at each point *q* in the path.

When the path is geodesic the 2 vectors are parallel, i.e.$\cos\left(\theta\right)=\left\langle\vec{n},\vec{N}\right\rangle=1$, the unit gradient *dq* is an *Extremal* of the quadratic $Q\left(dq\right)=-\left\langle dN\left(dq\right),dq\right\rangle$associated to the differential of the *Gauss Map* (8)$dN_{q}:T_{q}S\to T_{q}S$, a linear self-adjoint map operating on the tangent space to *S (*see Figure 1 above*)*. In our equation *Q* is obtained thorugh the *Hessian* (*H*) of$\left(r\circ f\right)^{task}$ which defines the distance metric for the given task. The leading eigenvector of this quadratic form is congruent with the gradient *dq*. The first eigenvalue of *H* coincides with the quadratic’s value at the *Extremal dq*.

**Second**, we show empirically that the hand paths can be well characterized as length minimizing curves, reparameterizable in time without affecting their conservation in both joint angle space and in the hand space. This means that their length is preserved. This along with previous experimental evidence from natural motions *(4, 6, 9-*11) motivated our use of a geometric optimal solution where the changes in temporal dynamics do not disrupt the uniqueness property of these curves being the minimal in length for each situation under study.


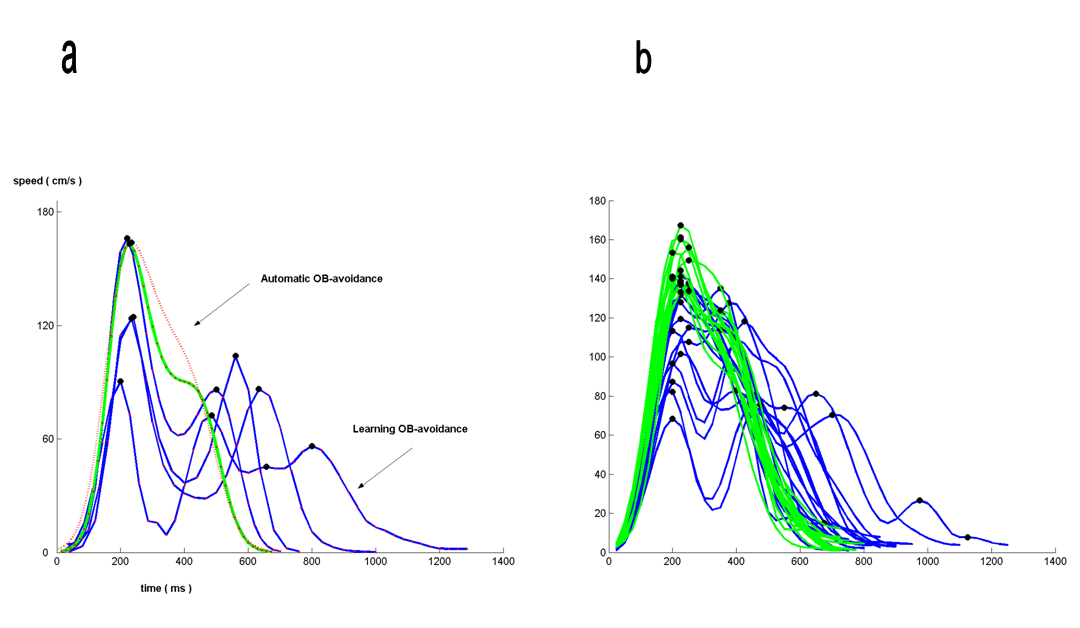


***SM Figure 3: Simulation of the timing to endow the geodesic curve with a speed profile and evolve the timing iteratively on a trial-by-trial fashion. (a) Simulated process with 6 iterations. Red curve is smooth and has a unique maximum so the iterations stop. (b) Actual trial-by-trial process from the hand trajectory data, blue are earlier trials, green are later trials. Black dots mark critical (maxima) points in the motion segments.***

To further simulate various timing profiles along the geodesic path we used a simple model. The model starts with a jerky speed profile from the data, denoted$speed^{\text{learning}_{1}}$. Key highlighted features used in the model include: the maximum speed value e.g.$\mu_{1}=150\frac{cm}{s}$ (similar to that of a typical 45 cm long straight reach), the time tau to reach that speed maximum, which we found to be highly consistent and characteristic of each location, and denoted here $\sigma_{1}=200ms$ in the Gaussian equation, and the initial movement duration (e.g. 1,300 ms which eventually shrinks to a consistent one of 600 ms).

Speed learning in the simulations evolved through several iterations, e.g. $k=6$, that changed the original broken profile into the final skewed-singled peak profile$speed^{\text{learning}_{6}}$. Iterations were defined $speed_{t_{i}}^{\text{learning}_{\text{k+1}}}=speed_{t_{i}}^{\text{learning}_{\text{k}}}\cdot speed_{t_{i}}^{\text{ideal}_{\text{k}}}$, and $speed_{t_{i}}^{\text{ideal}_{\text{k}}}=\left(\frac{e^{-\left[\frac{X-\mu_{k}}{2\sigma_{k}}\right]^{2}}}{\sigma_{k}\sqrt{2\pi}}\right)$ is the normal distribution in the variable *X* with mean$\mu$and variance$\sigma^{2}$. The variable *X* at each point $t_{i}$in the time interval $\left[0,t_{duration}\right]$comes from setting the peak speed at midway (as in straight reaches) and obtaining the profile corresponding to the time duration of the current iteration.

The value$speed_{t_{i}}^{\text{ideal}_{\text{k}}}$ was scaled by$speed_{t_{i}}^{\text{learning}_{\text{k}}}$, the speed magnitude at point $t_{i}$of the last updated OB-avoidance profile. For each pass$k=1\ldots6$, $\mu_{k}$is recomputed as the mean over *k* of the first significant maximum speed value and $\sigma_{k}$ as the time duration it takes to achieve it in the last updated temporal path. Each pass gives a different time partition of the interval with a speed magnitude for the curved OB-avoidance path at each point$t_{i}\in\left[0,t_{duration}\right]$. The magnitude of the first maximum speed value changes, the movement duration shortens, and the number of speed maxima decreases. Iterations stop when a single peak profile is reached. Importantly the distance delta traveled up to the peak velocity is a parameter in the metric expression of the gradient model.

**Third**, we morph the Riemannian length minimizing curves towards the Euclidean length-minimizing curve and study the distribution of area-perimeter ratios as the curves deform in each direction: back and forth from the particular case of Euclidean straight lines to the general cases of Riemannian straight lines SM Figure 4).


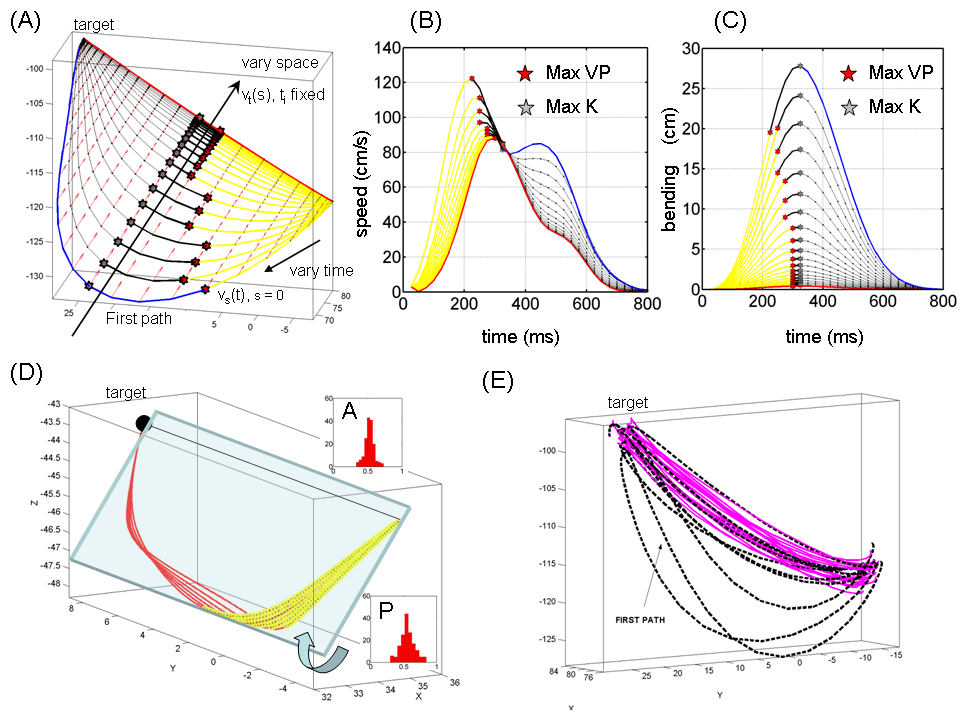


***SM Figure 4: Simulation of the de-adaptation process using variational principles. (A) Deformation of a path from the data towards the Euclidean geodesic. Yellow marks the first pulse, the distance traveled up to the peak velocity (marked with a red star). This is a parameter in the cost defining OB-avoidance (fully described in (4)). Black segment is the curve length traced between the maximum speed point and the maximum curvature point (marked with a grey star). (B) Resulting evolution of the timing along the curve. Blue speed profile is modeled after the data (using the parameters from the data trajectory). Red final iteration is unimodal. Stars representing the critical spatio-temporal landmarks correspond to those in (A). (C) Evolution of the path curvature when smoothly changing from one metric to the next and preserving the point in time of maximum curvature. (D) Curves generated with the gradient equation by wiggling the plane and setting the delta (yellow segment) to different values in the Cost. Distributions plotted in red of the area (A) and perimeter (P) ratios from the simulations. Similar symmetric ratios come from the distance-preserving cases where geodesics in X transform to geodesics in Q. (E) Actual de-adaptation trajectories from the data of one block. Black paths are the initial ones. Magenta paths are the ones that the system recovers later in the block. The first path of the block is marked.***

We modeled the de-adaptation process with variational principles (**SM Figure 4**): Arc-length parameterized paths that are generated using the gradient of the distance metric can be characterized as critical points of the length$L\left(c\right)=\int_{0}^{a} \left\|c'\left(s\right)\right\|ds$, or equivalently of the energy *(*12)$E\left(c\right)=\int_{0}^{a} \left\|c'\left(s\right)\right\|^{2}ds$for a curve $c:\left[0,a\right]\to M$. Starting at the first curved-residual path a proper variational family is obtained by varying along space $s\in\left(-\varepsilon,\varepsilon\right)$and time$t\in\left[0,a=t_{duration}\right]$. Given $\nu:\left(-\varepsilon,\varepsilon\right)\times\left[0,a\right]\to M$ with $E\left(s\right)=\int_{0}^{a} \left\|\frac{\partial\nu}{\partial t}\left(s,t\right)\right\|^{2}dt$, a time partition $0<t_{0}\ldots<t_{k+1},$ $i=0,\ldots,k$ such that the restriction of $\nu$to each $\left(-\varepsilon,\varepsilon\right)\times\left[t_{i},t_{i+1}\right]$is differentiable. For each $s\in\left(-\varepsilon,\varepsilon\right)$the parameterized differentiable curve $\nu_{s}:\left[0,a\right]\to M$given by $\nu\left(s,t\right)$is a curve in the variation, with $\nu_{0}\left(t\right)=c\left(t\right)$being the first curve and $\nu_{s}\left(t\right)$determining the family (**SM Figure 4**). A transversal curve in the variation is $\nu_{t}\left(s\right)=\nu\left(t,s\right)$with *t*-fixed, computed at$s=0$. This produces a piece-wise differentiable transversal vector field $\nu\left(t\right)=\frac{\partial\nu}{\partial s}\left(0,t\right)$ along$c\left(t\right)$(**SM Figure 4**). This transversal field gives rise to the variational family that converges from the transitional curved paths to the non-obstacle straighter reaching paths.

An ideal visual memory -a visually defined straight path- is assumed as the intended template to stop the deformation (morphing) process. For fixed *s*, starting at the first curved path in space $\nu_{s=0}\left(t\right)=c\left(t\right)$ with a non-unimodal speed profile, we vary along the time domain and generate the first transversal vector field $\nu\left(t\right)=\frac{\partial\nu}{\partial s}\left(0,t\right)$by projecting the points of the curved path onto the matching straight line joining the initial and target locations. The magnitude of each transversal vector is the distance to the matching point on the straight line divided by the path length of the current curved path along which the field is being built. The net effect for the next path $\nu_{s=1}\left(t\right)$ is (1) reduction in path length, (2) decrease in path curvature, and (3) shortening of the time duration. Iteratively building the transversal field guided by the matching ideal straight line of each $\nu_{s}\left(t\right)$ leads to the desired straight-reach path and moves towards a unimodal speed profile. Systematically simulating this morphing process across multiple targets and temporal profiles serves as a “synthetic arena” to further explore the empirical phenomena. We found that (1) whenever we used two geodesics -to start and to end the morphing process (as in **SM Figure 4**)- and regardless of different jerky tempos generated as in **SM Figure 3**, the final Euclidean stopping geodesic always converged to a speed profile with a single velocity peak; (2) This morphing process was smooth (had no singularities) and converged faster than the instances when the original curve was not a geodesic.

**References**

1. S. Gallot, D. Hulin, J. Lafontaine, *Riemannian geometry*. Universitext (Springer, Berlin ; New York, ed. 3rd, 2004), pp. xv, 322 p.

2. M. P. d. Carmo, *Differential geometry of curves and surfaces*. (Prentice-Hall, Englewood Cliffs, N.J., 1976), pp. viii, 503 p.

3. M. P. d. Carmo, *Riemannian geometry*. Mathematics. Theory & applications (Birkhäuser, Boston, 1992), pp. 300 p.

4. E. Torres, R. Andersen, *J Neurophysiol* **96**, 2613 (Nov, 2006).

5. E. B. Torres, D. Zipser, *J Neurophysiol* **88**, 2355 (Nov, 2002).

6. E. B. Torres, D. Zipser, *J Appl Physiol* **96**, 1978 (May, 2004).

7. C. Yu, Gu, X., Ballard, D. H. , paper presented at the *AAAI-04 Workshop on Supervisory Control of Learning and Adaptive Systems*, 2004.

8. M. Do Carmo, *Differential Geometry of Curves and Surfaces*. (Prentice Hall, 1976).

9. C. G. Atkeson, J. M. Hollerbach, *J Neurosci* **5**, 2318 (Sep, 1985).

10. K. C. Nishikawa, S. T. Murray, M. Flanders, *J Neurophysiol* **81**, 2582 (May, 1999).

11. E. Guigon, P. Baraduc, M. Desmurget, *J Neurophysiol* **97**, 331 (Jan, 2007).

12. S. Gallot, Hulin, D., Lafontaine, J., *Riemannian Geometry*. (Springer-Verlag, Berlin, Heidelberg, New York, London, Paris, Tokyo, Hong Kong, Barcelona, ed. Second, 1993).
